# Comparative effectiveness of tenofovir versus entecavir in patients with hepatitis B virus-related cirrhosis in Taiwan: a retrospective cohort study

**DOI:** 10.3389/fphar.2023.1301120

**Published:** 2023-12-19

**Authors:** Yu-Han Huang, Chuan-Wei Shen, Chung-Yu Chen, Ming-Jong Bair

**Affiliations:** ^1^ Department of Pharmacy, Pingtung Veterans General Hospital, Pingtung, Taiwan; ^2^ Master Program in Clinical Pharmacy, School of Pharmacy, Kaohsiung Medical University, Kaohsiung, Taiwan; ^3^ Department of Pharmacy, Kaohsiung Medical University Hospital, Kaohsiung, Taiwan; ^4^ Department of Medical Research, Kaohsiung Medical University Hospital, Kaohsiung, Taiwan; ^5^ Division of Gastroenterology, Department of Internal Medicine, Taitung Mackay Memorial Hospital, Taitung, Taiwan; ^6^ Department of Medicine, Mackay Medical College, New Taipei, Taiwan

**Keywords:** tenofovir, entecavir, effectiveness, hepatitis B virus, cirrhosis

## Abstract

**Background:** Tenofovir and entecavir demonstrated substantial effectiveness in the reversion of fibrosis and reversed cirrhosis in patients with hepatitis B virus (HBV)-related cirrhosis. However, there has not been a definitive conclusion regarding the association between entecavir and tenofovir on the risk of cirrhosis-related complications. Therefore, this study aimed to investigate the comparative effectiveness between tenofovir and entecavir in HBV-related cirrhosis patients.

**Methods:** This was a retrospective study using Taiwan’s Health Insurance Research Database. We enrolled newly diagnosed HBV-related cirrhosis patients who initiated entecavir and tenofovir between 2011 and 2019. Treatment groups were determined by the initial HBV antiviral medication prescribed. The primary composite outcome was the development of hepatocellular carcinoma (HCC), death from any causes, and liver transplantation. The secondary outcomes included all the individual components of the primary outcome. The incidence rate was calculated for each outcome for both treatment groups using the Fine–Gray subdistribution hazard models. Propensity score adjustment was used to balance treatment groups.

**Results:** A total of 7,316 propensity score-matched treatment-naïve patients and 3,524 propensity score-matched treatment-experienced patients were included. Within treatment-naïve patients, those receiving tenofovir showed significantly lower hazards of developing the composite outcome (HR, 0.79; *p* < 0.0001), hepatocellular carcinoma (HR, 0.86; *p* = 0.027), mortality (HR, 0.75; *p* < 0.0001), and liver transplantation (HR, 0.70; *p* = 0.0189) than those receiving entecavir. As for treatment-experienced patients, tenofovir was associated with a significantly lower risk of the composite outcome (HR, 0.82; *p* = 0.0033) and hepatocellular carcinoma (HR, 0.60; *p* < 0.0001), but it did not show a significantly different risk of all-cause mortality (HR, 0.93; *p* = 0.3374) or liver transplantation (HR, 1.17; *p* = 0.5112) compared to entecavir.

**Conclusion:** Tenofovir presented a significantly lower incidence of cirrhosis-related complications than entecavir in patients with hepatitis B virus-related cirrhosis. However, no statistically significant difference in death and liver transplantation was seen in treatment-experienced patients.

## Introduction

Cirrhosis is the leading cause of hepatocellular carcinoma (HCC) and results in approximately 1.16–1.32 million annual deaths globally (GBD, 2017 Cirrhosis Collaborators, 2020). Cirrhosis due to hepatitis B virus infection, namely, hepatitis B virus (HBV)-related cirrhosis, is responsible for over 50% of cirrhosis-related deaths in Asian nations (Sarin et al., 2020). In patients with HBV-related cirrhosis, clinicians would administer HBV antiviral drugs to suppress viral replication, reduce viral load, and thereby prevent cirrhosis progression and even reverse cirrhosis (Marcellin and Asselah, 2013; Calvaruso and Craxì, 2014; Rockey, 2016; Udompap and Kim, 2020).

Among the available nucleos(t)ide analogs (NAs), entecavir (ETV) and tenofovir (TDF/TAF) are recommended as first-line treatments for HBV-related cirrhosis considering their high antiviral efficacy and low rates of resistance (Sarin et al., 2016; European Association for the Study of the Liver, 2017; Terrault et al., 2018). As shown by previous randomized controlled trials, TDF/TAF and ETV demonstrated substantial effectiveness in the reversion of fibrosis and reversed cirrhosis in patients with HBV-related cirrhosis (Schiff et al., 2008; Yokosuka et al., 2010; Schiff et al., 2011; Marcellin et al., 2013).

Previous studies have indicated that TDF/TAF or ETV use may result in different effects on cirrhosis-related outcomes. The reason was that TDF/TAF belongs to the class of acyclic nucleoside phosphonates (ANPs) (De Clercq and Holý, 2005), and its structure differs from that of nucleoside analogs such as ETV. ANPs are characterized by prolonged action (De Clercq and Holý, 2005) and may exhibit better anti-HCC (Sato et al., 2006; Abushahba et al., 2010; Murata and Mizokami, 2023; Yang et al., 2023) and anti-HBV (Murata et al., 2020) effects. However, real-world evidence and experimental research regarding the comparative effectiveness between TDF/TAF and ETV in cirrhosis patients showed conflicting results (Choi et al., 2019; Papatheodoridis et al., 2020; Lee et al., 2021). Therefore, there has not been a definitive conclusion regarding the association between ETV and TDF/TAF on the risk of cirrhosis-related complications. Furthermore, there was a lack of evidence regarding the comparative effectiveness between TDF/TAF and ETV in treatment-experienced cirrhosis patients.

Therefore, this study aimed to investigate the hazards of cirrhosis-related complications, including HCC and liver transplantation, and mortality in patients with HBV-related cirrhosis receiving ETV and TDF/TAF.

## Materials and methods

### Study design and data sources

This retrospective cohort study was conducted using data from the National Health Insurance Research Database (NHIRD), which covered the healthcare data of approximately 100% of Taiwan’s population (National Health Insurance Administration, 2023a). The healthcare information in the database included that of diagnoses, treatments, operations, and prescription details. The study period was from 1 January 2010 to 31 December 2020. This study was approved by the Institutional Review Board (IRB) of Kaohsiung Medical University Hospital (IRB number: KMUHIRB-E(I)-20230042).

### Study population and exposure

Our study population included newly diagnosed HBV-related cirrhosis patients (adults), who had initiated ETV and TDF/TAF between 2011 and 2019. HBV-related cirrhosis was defined as chronic hepatitis B (CHB) diagnosed with cirrhosis after the initial CHB diagnosis. At least one inpatient visit or three outpatient visits were required to determine the number of CHB patients and for cirrhosis diagnosis. Diagnostic codes from the International Classification of Diseases, Ninth Revision, Clinical Modification (ICD-9-CM) and International Classification of Diseases, Tenth Revision, Clinical Modification (ICD-10-CM) were used to enroll HBV-related cirrhosis patients. The population entry date was defined as the date of the first diagnosis of cirrhosis. The baseline period was the time period within 1 year before the population entry date.

Patients who were below 20 years of age at the population entry date; had incomplete demographic information (including age, gender, or premium insurance); had a history of cirrhosis, liver transplantation, or HCC during the baseline period; or initiated ETV and TDF/TAF together were excluded from the study. Cirrhosis and liver transplantation were identified by the presence of ICD codes, while patients with HCC were defined by the presence of the ICD codes for HCC and inclusion in the Taiwan Cancer Registry long-form database (Kao et al., 2021).

Eligible patients were those with HBV-related cirrhosis who filled their first prescription for either ETV or TDF/TAF after the population entry date. Patients were divided into ETV or TDF/TAF groups based on the initial HBV antiviral medication prescribed after the population entry date. The index date was defined as the first day of receiving ETV or TDF/TAF following the population entry date. Follow-up began on the index date. Patients were stratified into the previously untreated (PUT) cohort and previously treated (PT) cohort (Supplementary eMethods 1) for the analysis.

### Study outcomes and follow-up

One primary outcome was evaluated: the composite outcome of HCC, liver transplantation, and all-cause death. Secondary outcomes were individual components of the primary outcome. The detailed definition of each outcome event is shown in Supplementary eMethods 2. Patients who had experienced the outcome event before the index date were excluded from the corresponding outcome analyses. Patients were followed up from the index date to the occurrence of the corresponding outcome, switching antiviral treatment, or the end date of the database (31 December 2020), whichever came first. Patients with discontinuation were censored until they switched treatment or re-initialized treatment. Discontinuation was defined as a gap of more than 30 days between the end of a prescription and the next. In each outcome analysis, patients were not censored if other outcomes (except for the corresponding outcome) had occurred earlier.

### Covariates and confounders

Patients’ baseline characteristics and medical information were retrieved from the database. The demographic information including age and gender was obtained from the most recent insurance record prior to the population entry date. Comorbidities were defined as diseases diagnosed at least once in an inpatient or twice in an outpatient setting within 1 year before the population entry date. Detailed information on comorbidities is summarized in Supplementary eTable S1. The Charlson Comorbidity Index (CCI) was used to quantify the comorbidity status of the included patients (Charlson et al., 1987). Co-medications being regarded as confounders were collected (Hayward and Weersink, 2020), and medications prescribed for a minimum of 28 days within the year before the population entry date were co-medications. The disease progression period and treatment gap period were retrieved. The disease progression period was defined as the period between the first CHB diagnosis and the population entry date. The treatment gap period was defined as the period from the population entry date to the index date.

### Propensity score methods

Two propensity score methods, namely, propensity score matching (PSM) and stabilized inverse probability of treatment weighting (IPTW), were used to generate comparable treatment groups before data analyses.

The PSM was performed using the 1:1 nearest-neighbor matching approach, with a caliper width set at 0.2 of the standard deviation of the logit of the propensity score (PS) (Austin, 2011a; Austin, 2011b). Confounders adjusted were age, gender, disease progression time, treatment gap duration, diabetes, hypertension, CCI, HCV/HDV/HEV co-infection, alcoholic cirrhosis, biliary cirrhosis, history of cirrhosis-related complications, and chronic kidney disease (CKD).

### Statistical analyses

HBV-related cirrhosis patients were stratified into PUT patients and PT patients to obtain results. In the baseline analysis, descriptive statistics were stratified by groups. Continuous variables were presented as mean and standard deviation (SD). Categorical variables were represented using the number (N) and percentage (%). To assess the balance in each covariate, standardized mean difference (SMD) was employed, with a value below 0.1 indicating negligible differences between the groups (Austin, 2009a; Austin, 2009b).

Fine–Gray subdistribution hazard models, accounting for the competing risk events of death and liver transplantation, were used to investigate subdistribution HRs with a 95% confidence interval (CI) for each outcome analysis (except for the composite outcome and all-cause death analysis because no competing risk events existed). The proportional hazard assumptions were evaluated before analyses. We conducted sensitivity analyses to evaluate the robustness of our findings. We used the negative control outcome, myocardial infarction, to indirectly evaluate whether potential confounders existed (Lipsitch et al., 2010).

A statistically significant difference was defined as a two-tailed probability value less than 0.05. Data management and statistical analyses were processed with SAS software version 9.4.

## Results

### Patient characteristics

**FIGURE 1:**
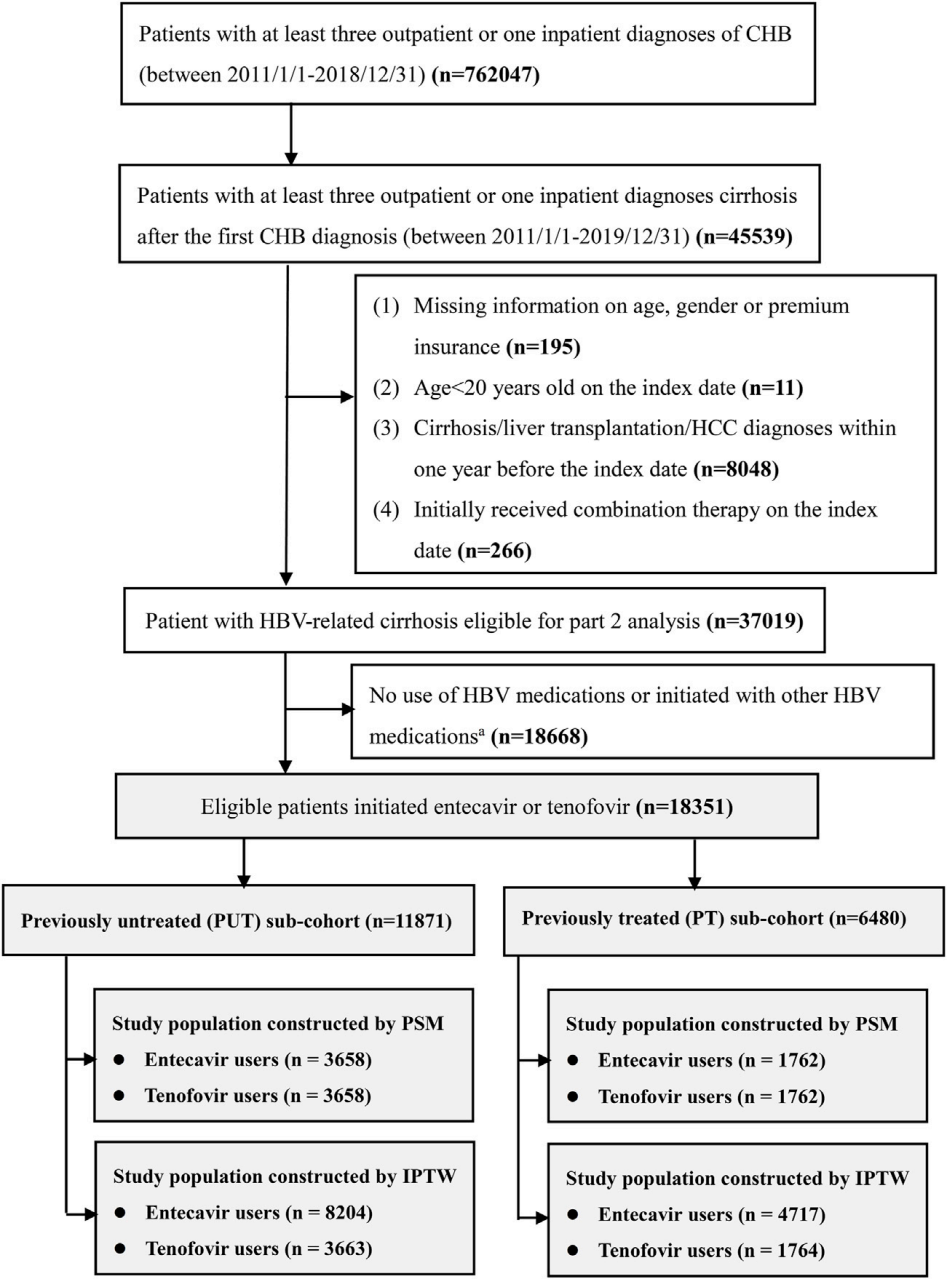
Flowchart of patients’ enrollment. CHB, chronic hepatitis B; HCC, hepatocellular carcinoma ^a^; other HBV medications include lamivudine, telbivudine, adefovir, and interferon.

The original study population contained 18,351 patients after applying inclusion and exclusion criteria. When PSM was used, 3,658 patients each were included in the ETV and TDF/TAF users in the PUT sub-cohort and 1,762 each were included in the ETV and TDF/TAF users in the PT sub-cohort. After applying stabilized IPTW, a weighted pseudopopulation consisted of 8,204 ETV users and 3,663 TDF/TAF users in the PUT sub-cohort and 4,717 ETV users and 1,764 TDF/TAF users in the PT sub-cohort. The enrollment process for the study population is illustrated in Figure 1. All patients in our study were included in the analysis of death outcome, and the baseline characteristics are presented in Table 1; Supplementary eTable S3. Overall, the mean age ranged from 54 to 57 years, and the majority were men (73%–77%). The mean disease progression period was 2.30–3.69 years. The baseline characteristics of patients for the analysis of the composite outcome, HCC, and liver transplantation are presented in Supplementary eTables S4–S9, respectively.

**TABLE 1:** Baseline characteristics of HBV-related cirrhosis patients within the PUT cohort after applying propensity score methods.

Characteristics	Population after PSM			Population after IPTW		
	ETV (*n* = 3,658)	TDF/TAF (*n* = 3,658)	ASMD^a^	ETV (*n* = 8,204)	TDF/TAF (*n* = 3,663)	ASMD^a^
Mean age (SD), y	55.14 (11.71)	55.05 (11.70)	0.008	56.74 (11.83)	56.70 (11.77)	0.003
Gender, *n* (%)						
Male	2,702 (73.87)	2,705 (73.95)	0.002	6,019 (73.37)	2,689 (73.42)	0.001
Female	956 (26.13)	953 (26.05)	0.002	2,185 (26.63)	973 (26.58)	0.001
Comorbidities, *n* (%)						
HCV co-infection	133 (3.64)	144 (3.94)	0.016	339 (4.13)	149 (4.08)	0.002
HDV co-infection	<3	<3	0.016	<3	<3	0.291
HEV co-infection	<3	<3	0.016	<3	<3	0.017
HIV co-infection	15 (0.41)	20 (0.55)	0.090	8 (0.1)	21 (0.58)	0.084
Alcoholic cirrhosis	91 (2.49)	88 (2.41)	0.005	205 (2.49)	91 (2.49)	0.000
Biliary cirrhosis	<3	<3	0.005	<3	<3	0.000
Hypertension	1,140 (31.16)	1,120 (30.62)	0.012	2,830 (34.50)	1,262 (34.45)	0.001
Hyperlipidemia	646 (17.66)	629 (17.2)	0.012	1,493 (18.20)	663 (18.1)	0.003
Diabetes	833 (22.77)	829 (22.66)	0.003	2092 (25.51)	938 (25.62)	0.003
Chronic kidney disease	85 (2.32)	95 (2.6)	0.018	377 (4.60)	165 (4.50)	0.005
History of complications, *n* (%)						
Ascites	107 (2.93)	106 (2.9)	0.002	295 (3.59)	128 (3.51)	0.005
Hepatic encephalopathy	405 (11.07)	396 (10.83)	0.008	965 (11.76)	435 (11.87)	0.003
EVB	50 (1.37)	51 (1.39)	0.002	116 (1.42)	53 (1.45)	0.003
Hepatorenal syndrome	<3	<3	0.014	11 (0.13)	4 (0.11)	0.006
Charlson Comorbidity Index						
Mean (SD)	1.43 (1.58)	1.38 (1.58)	0.025	1.59 (1.73)	1.59 (1.77)	0.038
Disease progression period (y)	2.51 (2.35)	2.46 (2.33)	0.019	2.30 (2.27)	2.30 (2.27)	0.002
Treatment gap period (y)	1.04 (1.73)	1.05 (1.72)	0.008	1.02 (1.71)	1.03 (1.70)	0.004
Co-medications, *n* (%)						
ACEIs/ARBs	709 (19.38)	661 (18.07)	0.034	1746 (21.28)	752 (20.54)	0.018
*β*-blockers	473 (12.93)	471 (12.88)	0.002	1,111 (13.54)	532 (14.51)	0.028
Non-selective	212 (5.80)	218 (5.96)	0.007	501 (6.11)	246 (6.71)	0.024
Selective	276 (7.55)	271 (7.41)	0.005	669 (8.15)	312 (8.51)	0.013
CCBs	610 (16.68)	585 (15.99)	0.018	1,481 (18.05)	672 (18.36)	0.008
Diuretics	498 (13.61)	434 (11.86)	0.052	1,253 (15.28)	511 (13.96)	0.037
Furosemide	138 (3.77)	100 (2.73)	0.059	393 (4.79)	131 (3.58)	0.061
Spironolactone	91 (2.49)	69 (1.89)	0.041	235 (2.86)	83 (2.28)	0.037
Insulin	112 (3.06)	110 (3.01)	0.003	324 (3.95)	130 (3.55)	0.021
Biguanide	532 (14.54)	525 (14.35)	0.005	1,269 (15.47)	578 (15.79)	0.009
Meglitinide	41 (1.12)	42 (1.15)	0.003	144 (1.76)	51 (1.39)	0.029
Sulfonylurea	424 (11.59)	376 (10.28)	0.042	1,008 (12.28)	420 (11.47)	0.025
*α*-glucosidase inhibitors	110 (3.01)	101 (2.76)	0.015	237 (2.89)	115 (3.13)	0.014
Thiazolidinediones	88 (2.41)	75 (2.05)	0.024	222 (2.69)	86 (2.36)	0.021
DPP-4 inhibitors	271 (7.41)	237 (6.48)	0.037	631 (7.70)	266 (7.26)	0.016
SGLT2 inhibitors	20 (0.55)	34 (0.93)	0.045	42 (0.51)	35 (0.95)	0.052
GLP1 agonists	8 (0.22)	5 (0.14)	0.019	10 (0.12)	4.82 (0.13)	0.004
Statin	369 (10.09)	358 (9.79)	0.010	868 (10.58)	398 (10.87)	0.009
Fibrates	86 (2.35)	79 (2.16)	0.013	180 (2.20)	79.49 (2.17)	0.002
Silymarin	871 (23.81)	884 (24.17)	0.008	1933 (23.56)	911 (24.86)	0.030

IPTW, inverse probability of treatment weighting; ASMD, absolute standardized mean difference; ETV, entecavir; TDF/TAF, tenofovir disoproxil fumarate/tenofovir alafenamide fumarate; HCV, hepatitis C virus; HDV, hepatitis D virus; HIV, hepatitis I virus; EVB, esophageal varices with bleeding; ACEIs, angiotensin-converting enzyme inhibitors; ARBs, angiotensin II receptor blockers; CCBs, calcium-channel blockers; SGLT2, sodium-glucose cotransporter-2; GLP1, glucagon-like peptide-1; y: year.

^a^ The absolute standardized mean difference less than 0.1 indicates well-balanced between groups.

### Hazards of developing cirrhosis-related outcomes in the PUT cohort

**FIGURE 2:**
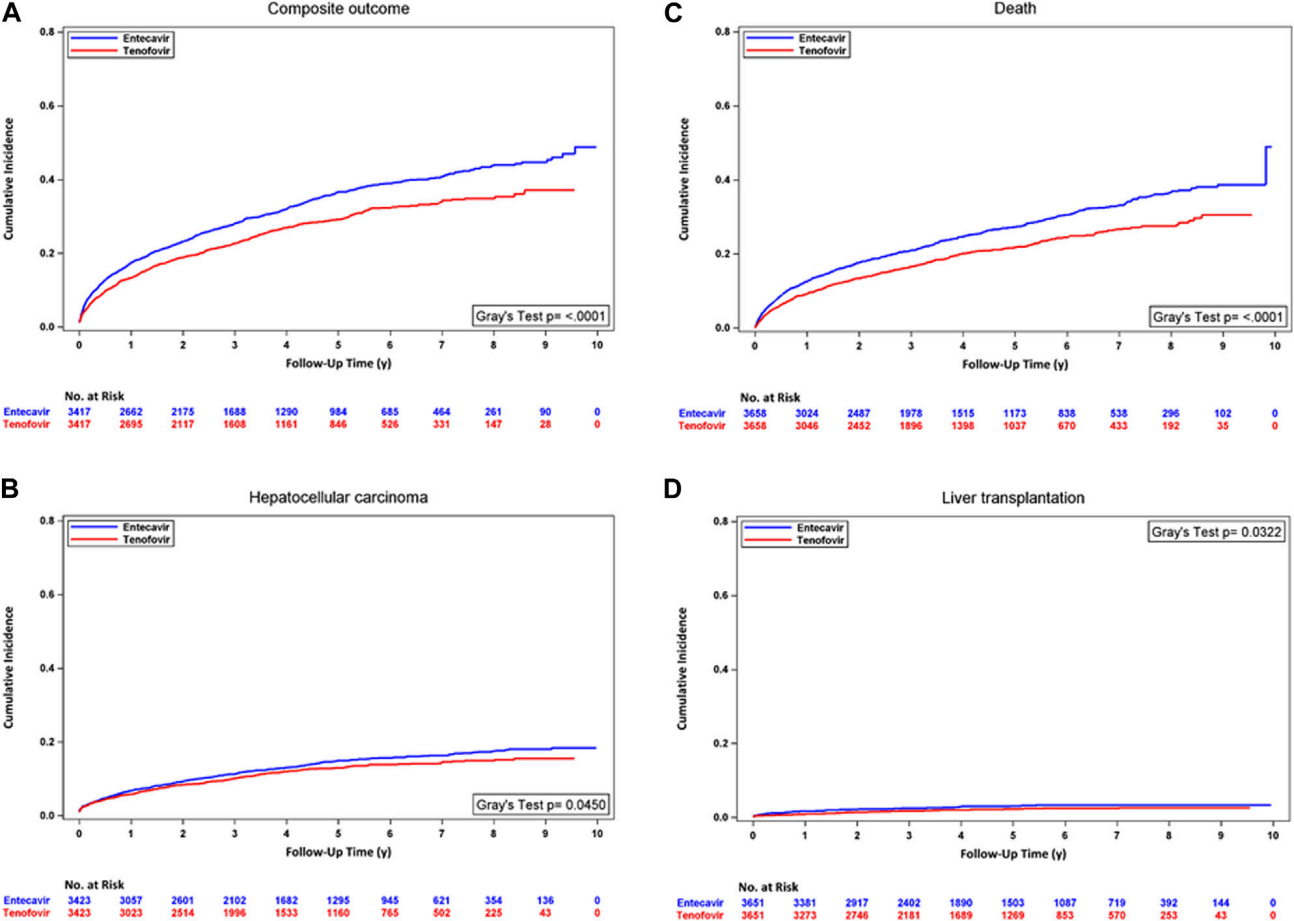
Cumulative incidence curves for TDF/TAF users versus ETV users within PUT cohorts after PSM. **(A)** Composite outcome, **(B)** hepatocellular carcinoma, **(C)** death, and **(D)** liver transplantation.

In the analyses with PSM, the incidence rate of the composite outcome, HCC, and mortality was significantly lower in the TDF/TAF users. TDF/TAF showed significantly lower hazards of developing the composite outcome [HR, 0.78 (95% CI, 0.72 to 0.85); *p* < 0.0001], HCC [HR, 0.87 (95% CI, 0.76 to 0.99); *p* = 0.0396], mortality [HR, 0.76 (95% CI, 0.68 to 0.83); *p* < 0.0001], and liver transplantation [HR, 0.72 (95% CI, 0.53 to 0.97); *p* = 0.0327] in unadjusted analysis accounting for competing risk. After adjusting for baseline confounders, similarly lower hazards of developing the composite outcome, HCC, mortality, and liver transplantation were seen in TDF/TAF users (Table 2 Panel A). The differences in cumulative incidence curves between treatment groups within the PUT cohort for four outcomes are shown in Figure 2. In the analyses with stabilized IPTW, similar hazards of the lower composite outcome, mortality, and liver transplantation were found in TDF/TAF users than in ETV users (Table 2 Panel B; Supplementary eFigure S1).

**TABLE 2:** Clinical outcomes within PUT patients after applying propensity score methods.

Panel A. Population after PSM								
Outcome^a^	Patients, *n*	Events, *n*	PY	Rate^b^ (95% CI)	csHR^c^ (95% CI)	*p*-value	asHR^d^ (95% CI)	*p*-value
Composite outcome								
Tenofovir	3,417	850	11,004	7.72 (7.21–8.26)	0.78 (0.72–0.85)	<0.0001	0.79 (0.72–0.86)	<0.0001
Entecavir	3,417	1,124	11,838	9.49 (8.95–10.07)	1.00 (reference)		1.00 (reference)	
Hepatocellular carcinoma								
Tenofovir	3,423	579	13,368	4.33 (3.99–4.70)	0.87 (0.76–0.99)	0.0396	0.86 (0.75–0.98)	0.027
Entecavir	3,423	748	14,438	5.18 (4.82–5.57)	1.00 (reference)		1.00 (reference)	
Death								
Tenofovir	3,658	686	12,877	5.33 (4.94–5.74)	0.76 (0.68–0.83)	< 0.0001	0.75 (0.67–0.82)	< 0.0001
Entecavir	3,658	941	13,668	6.88 (6.45–7.34)	1.00 (reference)		1.00 (reference)	
Liver transplantation								
Tenofovir	3,651	80	14,623	0.55 (0.43–0.68)	0.72 (0.53–0.97)	0.0327	0.70 (0.51–0.94)	0.0189
Entecavir	3,651	105	16,245	0.65 (0.53–0.78)	1.00 (reference)		1.00 (reference)	

Panel B. Population after IPTW								
Outcome^a^	Patients, *n*	Events, *n*	PY	Rate^b^ (95% CI)	cHR^c^ (95% CI)	*p*-value	aHR^d^ (95% CI)	*p*-value
Composite outcome								
Tenofovir	3,420	919	10,867	8.46 (7.92–9.02)	0.78 (0.72–0.84)	<0.0001	0.79 (0.73–0.85)	<.0001
Entecavir	7,464	2,653	25,361	10.46 (10.07–10.87)	1.00 (reference)		1.00 (reference)	
Hepatocellular carcinoma								
Tenofovir	3,427	621	13,471	4.61 (4.25–4.99)	0.93 (0.83–1.04)	0.182	0.92 (0.82–1.03)	0.1429
Entecavir	7,496	1,675	32,281	5.19 (4.94–5.44)	1.00 (reference)		1.00 (reference)	
Death								
Tenofovir	3,663	754	12,830	5.88 (5.47–6.31)	0.77 (0.71–0.84)	< 0.0001	0.77 (0.71–0.84)	< 0.0001
Entecavir	8,204	2,277	30,855	7.38 (7.08–7.69)	1.00 (reference)		1.00 (reference)	
Liver transplantation								
Tenofovir	3,655	79	14,804	0.53 (0.42–0.67)	0.67 (0.51–0.88)	0.0038	0.66 (0.50–0.87)	0.0028
Entecavir	8,160	247	37,226	0.66 (0.58–0.75)	1.00 (reference)		1.00 (reference)	

Abbreviations: PSM, propensity score matching; PY, person-year; cHR, crude hazard ratio; aHR, adjusted hazard ratio.

^a^ Patients who had already encountered the relevant outcome before the index date were excluded in every outcome analysis.

^b^ Rate was determined by dividing the number of events by the total person-years and presented as per 100 person-years.

^c^ Crude HR was calculated by the subdistribution COX proportional hazards model.

^d^ Adjusted HR was calculated by the subdistribution COX proportional hazards model adjusted for all variables.

### Hazards of developing cirrhosis-related outcomes in the PT cohort

**FIGURE 3:**
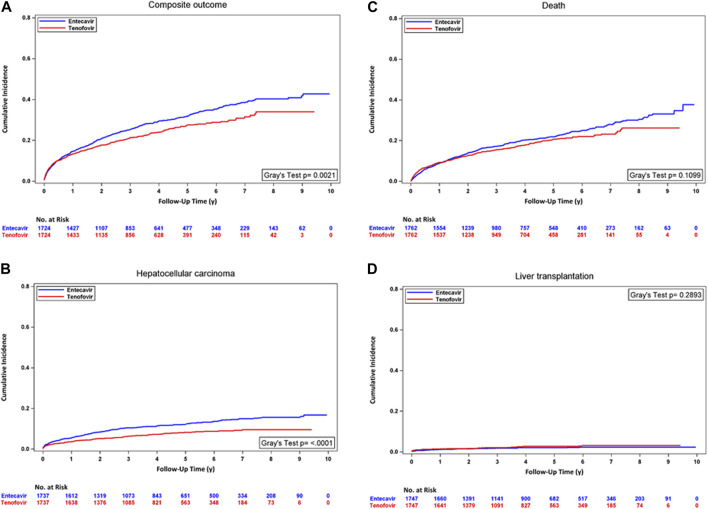
Cumulative incidence curves for TDF/TAF users versus ETV users within PT cohorts after PSM. **(A)** Composite outcome, **(B)** hepatocellular carcinoma, **(C)** death, and **(D)** liver transplantation.

In the analyses with PSM, the incidence rate of HCC was significantly lower in the TDF/TAF users. In unadjusted analysis accounting for competing risk, TDF/TAF showed significantly lower hazards of developing composite outcomes [HR, 0.81 (95% CI, 0.71 to 0.93); *p* = 0.0021] and HCC [HR, 0.61 (95% CI, 0.49 to 0.76); *p* < 0.0001]. TDF/TAF was associated with a lower incidence rate of death, but the result did not achieve statistical significance. After adjusting for baseline confounders, similarly lower hazards of developing composite outcomes and HCC were seen in TDF/TAF users. The risks of death and developing transplantation were not statistically different between the two groups (Supplementary eTable S10 Panel A). The cumulative incidence curves between treatment groups within the PT cohort for four outcomes are shown in Figure 3.

In the analyses with stabilized IPTW, similarly lower incidence rates of the composite outcome and HCC were seen in patients treated with TDF/TAF. TDF/TAF was associated with a lower incidence rate of mortality, but the result did not achieve statistical significance. The univariate and multivariate analyses accounting for competing risk events showed a similar trend of lower composite outcome and HCC hazards in TDF/TAF users than in ETV users. The risks of death and developing transplantation were not statistically different between the two groups (Supplementary eTable S10 Panel B; Supplementary eFigure S2).

### Results of sensitivity analysis

Regarding the analysis for the negative control outcome, the outcome did not show a significant association with TDF/TAF treatment (Supplementary eTable S11).

## Discussion

Our study demonstrated a significantly reduced risk of developing cirrhosis-related complications among TDF/TAF users, consistent with previous studies suggesting a lower risk of HCC in individuals with HBV-related cirrhosis who received TDF/TAF than those receiving ETV (Choi et al., 2019). The negative control outcome, namely, MI, supported the conclusion that the lower hazards of cirrhosis-related outcomes and death in TDF/TAF compared to ETV were robust.

Our study could not determine the exact mechanism underlying the better outcomes with TDF/TAF treatment. However, several reasons might explain our findings. First, TDF/TAF might show superior virologic response profiles compared to ETV, as presented in previous studies (Koike et al., 2018; Chen et al., 2019; Choi et al., 2019; Choi et al., 2021). These better virologic outcomes might lead to different levels of effectiveness in preventing cirrhosis-associated complications between TDF/TAF and ETV therapy. Second, the antitumor effects of TDF/TAF have been reported. The reason was that higher interferon-λ3 levels were induced by ANPs (such as TDF/TAF), but not by nucleoside analogs (such as ETV) (Sato et al., 2006; Abushahba et al., 2010; Murata and Mizokami, 2023; Yang et al., 2023). Interferon-λ3 demonstrated potent antitumor effects in murine cancer models, including HCC (Sato et al., 2006; Abushahba et al., 2010; Murata and Mizokami, 2023; Yang et al., 2023). The antitumor activity might explain the differences in risks in developing outcomes between TDF/TAF and ETV. Third, TDF/TAF was anticipated to generate favorable immune responses toward anti-HBV effects. As presented by Murata et al. (2020), TDF/TAF could inhibit interleukin (IL)-10 production and thereby promote the release of IL-12 and tumor necrosis factor (TNF)-α, which was not observed in ETV. Suppressed IL-10 and increased IL-12 would stimulate T cells and NK cells to induce IFN-γ (Henry et al., 2008; Smith et al., 2018). Both IFN-γ and TNF-α promoted anti-HBV effects by inhibiting HBV replication and decreasing HBV covalently closed circular DNA (cccDNA) levels (Cavanaugh et al., 1997; Rehermann and Bertoletti, 2015; Xia et al., 2016).

In the PUT cohort after propensity score matching methods, TDF/TAF showed a significantly lower rate in each outcome. However, TDF/TAF was significantly associated with a lower hazard in the composite outcome and HCC, but not in death or liver transplantation. The inconsistent results among outcomes might be explained as follows: the lack of difference in incidence of death can be attributed to a higher proportion of patients in the ETV groups experiencing deaths unrelated to HCC, compared to the TDF/TAF groups (data not shown). No difference in incidence of liver transplantation represented that most patients received liver transplants because of complications of decompensation rather than HCC (data not shown) (European Association for the Study of the Liver, 2018).

To date, only a few real-world studies have compared cirrhosis-related outcomes between TDF/TAF and ETV in HBV-related cirrhosis patients (Choi et al., 2019; Papatheodoridis et al., 2020). However, real-world evidence investigating the comparative effectiveness between TDF/TAF and ETV in Taiwanese patients with HBV-related cirrhosis was limited. Furthermore, the evidence comparing cirrhosis-related outcomes within treatment-experienced cirrhosis patients was scarce. Our study successfully addresses the current knowledge gap.

### Strengths and limitations

The main strengths of our study were as follows: this was a large-scale cohort study using the NHIRD to describe patients’ characteristics and the novel findings that comprehensively evaluated comparative effectiveness between TDF/TAF and ETV in Taiwanese HBV-related cirrhosis patients. Additionally, our findings were consistent with those of a previous cohort (Choi et al., 2019). Moreover, our study addressed the knowledge gap and provided information with comparative effectiveness evidence in patients with prior exposure to NA. Furthermore, our conclusion remained consistent across different propensity score methods and sensitivity analyses.

We acknowledge that some limitations remain in this study. First, HBV-related (e.g., HBV viral load and HBeAg status), liver function-related (e.g., AST and ALT), HCC-related (e.g., *α*-fetoprotein, family history of HCC, smoking status, alcohol status, and BMI), and cirrhosis-related (platelet count, bilirubin, albumin, prothrombin time, serum creatinine, and fibrosis markers) lab data and Chinese medicine exposure data could not be obtained in our database (Hsu et al., 2014; Chen et al., 2017; Zhang et al., 2021; Kanwal et al., 2023). For HBV-related and liver function-related lab data, the ETV and TDF/TAF could continue to be reimbursed regardless of HBV viral load, HBeAg status, or results of liver function tests in HBV-related cirrhosis patients under the NHI payment guidelines (National Health Insurance Administration, 2023b). Therefore, the absence of information would not substantially affect our findings because the missing information was unlikely to induce treatment selection bias. However, the lack of cirrhosis-related information could impact our ability to assess the severity of liver cirrhosis and hepatic failure. This could misidentify individuals without cirrhosis as having cirrhosis, and *vice versa*. In addition, the lack of HCC-related data was an unmeasured confounder in our study, which might influence our estimated results. Second, we used ICD codes to identify cirrhosis patients, which hindered our ability to accurately determine cirrhosis status. The generation of misclassification bias resulted from the absence of information concerning diagnostic procedures for cirrhosis in clinical practice (for example, liver biopsy, ultrasound, CT, MRI, and liver stiffness evaluation) (RadiologyInfo, 2022). Third, despite the use of propensity score methods to address confounding variables, unknown or unmeasured confounders might still exist. Fourth, there were potential reasons that would induce selection bias between treatment groups. Given that ETV had been approved 3 years before TDF/TAF, ETV users tended to be older and have more advanced diseases than TDF/TAF users. This “patient warehousing” phenomenon was similarly observed in previous studies (Lok et al., 2016; Hsu et al., 2020; Yip et al., 2020). Moreover, there were a few additional potential explanations for the relatively younger age and milder liver disease of TDF/TAF patients. One reason could be the preference for TDF/TAF among young women of childbearing age due to its safety during pregnancy. Additionally, concerns regarding renal toxicity and osteoporosis might lead to the avoidance of TDF in the elderly population (Sarin et al., 2016; European Association for the Study of the Liver, 2017; Terrault et al., 2018). Nonetheless, because our study was an active comparison design with similar indications, the misclassification population, difference in baseline characteristics, and other unmeasured confounders could be reduced (Yoshida et al., 2015). Fifth, our study used data from the NHIRD; therefore, it is necessary to conduct further studies to validate whether our findings could be extrapolated to other countries or regions.

Our study provided updated information regarding the comparative effectiveness between ETV and TDF/TAF. Further studies could evaluate the comparative cost-effectiveness between two treatments to guide the optimal distribution of healthcare system resources.

## Conclusion

TDF/TAF treatment was associated with a significantly lower risk of cirrhosis-related complications, and mortality, in patients with HBV-related cirrhosis compared with ETV treatment. However, no statistically significant difference in death and liver transplantation was seen in treatment-experienced patients. Further studies are necessary to ensure the replicability of our findings.

## Data Availability

The data analyzed in this study are subject to the following licenses/restrictions: C-YC had full access to all the data in the study and takes responsibility for the integrity of the data and the accuracy of the data analysis. Data are available from the National Health Insurance Research Database (NHIRD), published by the Bureau of National Health Insurance (BNHI) of the Ministry of Health and Welfare. Owing to the legal restrictions imposed by the Government of Taiwan related to the Personal Information Protection Act, the database cannot be made publicly available. The conclusions presented in this study are those of the authors and do not necessarily reflect the views of the BNHI, the Ministry of Health and Welfare. Requests to access these datasets should be directed to C-YC jk2975525@hotmail.com.

## References

[B1] Abushahba W. Balan M. Castaneda I. Yuan Y. Reuhl K. Raveche E. (2010). Antitumor activity of type I and type III interferons in BNL hepatoma model. CII 59 (7), 1059–1071. 10.1007/s00262-010-0831-3 20217081 PMC4699672

[B2] Austin P. C. (2009a). Using the standardized difference to compare the prevalence of a binary variable between two groups in observational research. Commun. Statistics - Simul. Comput. 38 (6), 1228–1234. 10.1080/03610910902859574

[B3] Austin P. C. (2009b). Balance diagnostics for comparing the distribution of baseline covariates between treatment groups in propensity-score matched samples. Statistics Med. 28 (25), 3083–3107. 10.1002/sim.3697 PMC3472075 19757444

[B4] Austin P. C. (2011a). An introduction to propensity score methods for reducing the effects of confounding in observational studies. Multivar. Behav. Res. 46 (3), 399–424. 10.1080/00273171.2011.568786 PMC3144483 21818162

[B5] Austin P. C. (2011b). Optimal caliper widths for propensity-score matching when estimating differences in means and differences in proportions in observational studies. Pharm. Stat. 10 (2), 150–161. 10.1002/pst.433 20925139 PMC3120982

[B6] Calvaruso V. Craxì A. (2014). Regression of fibrosis after HBV antiviral therapy. Is cirrhosis reversible? Liver international. official J. Int. Assoc. Study Liver 34 (1), 85–90. 10.1111/liv.12395 24373083

[B7] Cavanaugh V. J. Guidotti L. G. Chisari F. V. (1997). Interleukin-12 inhibits hepatitis B virus replication in transgenic mice. J. virology 71 (4), 3236–3243. 10.1128/JVI.71.4.3236-3243.1997 9060687 PMC191456

[B8] Charlson M. E. Pompei P. Ales K. L. MacKenzie C. R. (1987). A new method of classifying prognostic comorbidity in longitudinal studies: development and validation. J. chronic Dis. 40 (5), 373–383. 10.1016/0021-9681(87)90171-8 3558716

[B9] Chen C. H. Lee C. M. Lai H. C. Hu T. H. Su W. P. Lu S. N. (2017). Prediction model of hepatocellular carcinoma risk in Asian patients with chronic hepatitis B treated with entecavir. Oncotarget 8 (54), 92431–92441. 10.18632/oncotarget.21369 29190928 PMC5696194

[B10] Chen M. B. Wang H. Zheng Q. H. Zheng X. W. Fan J. N. Ding Y. L. (2019). Comparative efficacy of tenofovir and entecavir in nucleos(t)ide analogue-naive chronic hepatitis B: a systematic review and meta-analysis. PloS one 14 (11), e0224773. 10.1371/journal.pone.0224773 31751366 PMC6872143

[B11] Choi J. Kim H. J. Lee J. Cho S. Ko M. J. Lim Y. S. (2019). Risk of hepatocellular carcinoma in patients treated with entecavir vs tenofovir for chronic hepatitis B: a Korean nationwide cohort study. JAMA Oncol. 5 (1), 30–36. 10.1001/jamaoncol.2018.4070 30267080 PMC6439769

[B12] Choi W. M. Choi J. Lim Y. S. (2021). Effects of tenofovir vs entecavir on risk of hepatocellular carcinoma in patients with chronic HBV infection: a systematic review and meta-analysis. Clin. gastroenterology hepatology 19 (2), 246–258.e9. 10.1016/j.cgh.2020.05.008 32407970

[B13] De Clercq E. Holý A. (2005). Acyclic nucleoside phosphonates: a key class of antiviral drugs. Nat. Rev. Drug Discov. 4 (11), 928–940. 10.1038/nrd1877 16264436

[B14] European Association for the Study of the Liver (2017). EASL 2017 Clinical Practice Guidelines on the management of hepatitis B virus infection. J. Hepatol. 67 (2), 370–398. 10.1016/j.jhep.2017.03.021 28427875

[B15] European Association for the Study of the Liver (2018). EASL Clinical Practice Guidelines for the management of patients with decompensated cirrhosis. J. Hepatol. 69 (2), 406–460. 10.1016/j.jhep.2018.03.024 29653741

[B16] GBD 2017 Cirrhosis Collaborators (2020). The global, regional, and national burden of cirrhosis by cause in 195 countries and territories, 1990-2017: a systematic analysis for the Global Burden of Disease Study 2017. lancet Gastroenterology hepatology 5 (3), 245–266. 10.1016/S2468-1253(19)30349-8 31981519 PMC7026710

[B17] Hayward K. L. Weersink R. A. (2020). Improving medication-related outcomes in chronic liver disease. Hepatol. Commun. 4 (11), 1562–1577. 10.1002/hep4.1612 33163829 PMC7603526

[B18] Henry C. J. Ornelles D. A. Mitchell L. M. Brzoza-Lewis K. L. Hiltbold E. M. (2008). IL-12 produced by dendritic cells augments CD8+ T cell activation through the production of the chemokines CCL1 and CCL17. J. Immunol. Baltim. Md 1950) 181 (12), 8576–8584. 10.4049/jimmunol.181.12.8576 PMC2716729 19050277

[B19] Hsu Y. C. Wong G. L. Chen C. H. Peng C. Y. Yeh M. L. Cheung K. S. (2020). Tenofovir versus entecavir for hepatocellular carcinoma prevention in an international consortium of chronic hepatitis B. Am. J. gastroenterology 115 (2), 271–280. 10.14309/ajg.0000000000000428 31634265

[B20] Hsu Y. C. Wu C. Y. Lane H. Y. Chang C. Y. Tai C. M. Tseng C. H. (2014). Determinants of hepatocellular carcinoma in cirrhotic patients treated with nucleos(t)ide analogues for chronic hepatitis B. J. Antimicrob. Chemother. 69 (7), 1920–1927. 10.1093/jac/dku041 24576950

[B21] Kanwal F. Khaderi S. Singal A. G. Marrero J. A. Loo N. Asrani S. K. (2023). Risk factors for HCC in contemporary cohorts of patients with cirrhosis. Hepatology 77 (3), 997–1005. 10.1002/hep.32434 35229329 PMC9433461

[B22] Kao C. W. Chiang C. J. Lin L. J. Huang C. W. Lee W. C. Lee M. Y. (2021). Accuracy of long-form data in the Taiwan cancer registry. J. Formos. Med. Assoc. = Taiwan yi zhi 120 (11), 2037–2041. 10.1016/j.jfma.2021.04.022 34020856

[B23] Koike K. Suyama K. Ito H. Itoh H. Sugiura W. (2018). Randomized prospective study showing the non-inferiority of tenofovir to entecavir in treatment-naïve chronic hepatitis B patients. Hepatology Res. 48 (1), 59–68. 10.1111/hepr.12902 28374496

[B24] Lee S. W. Kim S. M. Hur W. Kang B. Y. Lee H. L. Nam H. (2021). Tenofovir disoproxil fumarate directly ameliorates liver fibrosis by inducing hepatic stellate cell apoptosis via downregulation of PI3K/Akt/mTOR signaling pathway. PloS one 16 (12), e0261067. 10.1371/journal.pone.0261067 34879114 PMC8654182

[B25] Lipsitch M. Tchetgen Tchetgen E. Cohen T. (2010). Negative controls: a tool for detecting confounding and bias in observational studies. Epidemiol. Camb. Mass) 21 (3), 383–388. 10.1097/EDE.0b013e3181d61eeb PMC3053408 20335814

[B26] Lok A. S. McMahon B. J. Brown R. S. Jr. Wong J. B. Ahmed A. T. Farah W. (2016). Antiviral therapy for chronic hepatitis B viral infection in adults: a systematic review and meta-analysis. Hepatology 63 (1), 284–306. 10.1002/hep.28280 26566246

[B27] Marcellin P. Asselah T. (2013). Long-term therapy for chronic hepatitis B: hepatitis B virus DNA suppression leading to cirrhosis reversal. J. gastroenterology hepatology 28 (6), 912–923. 10.1111/jgh.12213 23573915

[B28] Marcellin P. Gane E. Buti M. Afdhal N. Sievert W. Jacobson I. M. (2013). Regression of cirrhosis during treatment with tenofovir disoproxil fumarate for chronic hepatitis B: a 5-year open-label follow-up study. Lancet (London, Engl. 381 (9865), 468–475. 10.1016/S0140-6736(12)61425-1 23234725

[B29] Murata K. Mizokami M. (2023). Possible biological mechanisms of entecavir versus tenofovir disoproxil fumarate on reducing the risk of hepatocellular carcinoma. J. gastroenterology hepatology 38 (5), 683–691. 10.1111/jgh.16178 36918402

[B30] Murata K. Tsukuda S. Suizu F. Kimura A. Sugiyama M. Watashi K. (2020). Immunomodulatory mechanism of acyclic nucleoside phosphates in treatment of hepatitis B virus infection. Hepatology 71 (5), 1533–1545. 10.1002/hep.30956 31529730

[B31] National Health Insurance Administration (2023b). Payment guidelines for antimicrobial agents. Taiwan: Ministry of Health and Welfare. Available at: https://www.nhi.gov.tw/Content_List.aspx?n=E70D4F1BD029DC37&topn=5FE8C9FEAE863B46.

[B32] National Health Insurance Administration (2023a). NHI profile. Available at: https://www.nhi.gov.tw/English/Content_List.aspx?n=8FC0974BBFEFA56D&topn=ED4A30E51A609E49.

[B33] Papatheodoridis G. V. Dalekos G. N. Idilman R. Sypsa V. Van Boemmel F. Buti M. (2020). Similar risk of hepatocellular carcinoma during long-term entecavir or tenofovir therapy in Caucasian patients with chronic hepatitis B. J. Hepatol. 73 (5), 1037–1045. 10.1016/j.jhep.2020.06.011 32553667

[B34] RadiologyInfo (2022). Cirrhosis of the liver. Available at: https://www.radiologyinfo.org/en/info/cirrhosisliver.

[B35] Rehermann B. Bertoletti A. (2015). Immunological aspects of antiviral therapy of chronic hepatitis B virus and hepatitis C virus infections. Hepatology 61 (2), 712–721. 10.1002/hep.27323 25048716 PMC4575407

[B36] Rockey D. C. (2016). Liver fibrosis reversion after suppression of hepatitis B virus. Clin. liver Dis. 20 (4), 667–679. 10.1016/j.cld.2016.06.003 27742006 PMC6438202

[B37] Sarin S. K. Kumar M. Eslam M. George J. Al Mahtab M. Akbar S. M. F. (2020). Liver diseases in the asia-pacific region: a lancet gastroenterology & hepatology commission. lancet Gastroenterology hepatology. 5 (2), 167–228. 10.1016/S2468-1253(19)30342-5 31852635 PMC7164809

[B38] Sarin S. K. Kumar M. Lau G. K. Abbas Z. Chan H. L. Chen C. J. (2016). Asian-Pacific clinical practice guidelines on the management of hepatitis B: a 2015 update. Hepatol. Int. 10 (1), 1–98. 10.1007/s12072-015-9675-4 PMC4722087 26563120

[B39] Sato A. Ohtsuki M. Hata M. Kobayashi E. Murakami T. (2006). Antitumor activity of IFN-lambda in murine tumor models. J. Immunol. Baltim. Md 1950) 176 (12), 7686–7694. 10.4049/jimmunol.176.12.7686 16751416

[B40] Schiff E. Simsek H. Lee W. M. Chao Y. C. Sette H. Jr. Janssen H. L. (2008). Efficacy and safety of entecavir in patients with chronic hepatitis B and advanced hepatic fibrosis or cirrhosis. Am. J. gastroenterology 103 (11), 2776–2783. 10.1111/j.1572-0241.2008.02086.x 18721244

[B41] Schiff E. R. Lee S. S. Chao Y. C. Kew Yoon S. Bessone F. Wu S. S. (2011). Long-term treatment with entecavir induces reversal of advanced fibrosis or cirrhosis in patients with chronic hepatitis B. Clinical gastroenterology and hepatology: the official clinical practice. J. Am. Gastroenterological Assoc. 9 (3), 274–276. 10.1016/j.cgh.2010.11.040 21145419

[B42] Smith L. K. Boukhaled G. M. Condotta S. A. Mazouz S. Guthmiller J. J. Vijay R. (2018). Interleukin-10 directly inhibits CD8(+) T cell function by enhancing N-glycan branching to decrease antigen sensitivity. Immunity 48 (2), 299–312. 10.1016/j.immuni.2018.01.006 29396160 PMC5935130

[B43] Terrault N. A. Lok A. S. F. McMahon B. J. Chang K. M. Hwang J. P. Jonas M. M. (2018). Update on prevention, diagnosis, and treatment of chronic hepatitis B: AASLD 2018 hepatitis B guidance. Hepatology 67 (4), 1560–1599. 10.1002/hep.29800 29405329 PMC5975958

[B44] Udompap P. Kim W. R. (2020). Development of hepatocellular carcinoma in patients with suppressed viral replication: changes in risk over time. Clin. liver Dis. 15 (2), 85–90. 10.1002/cld.904 PMC7098665 32226623

[B45] Xia Y. Stadler D. Lucifora J. Reisinger F. Webb D. Hösel M. (2016). Interferon-γ and tumor necrosis factor-α produced by T cells reduce the HBV persistence form, cccDNA, without cytolysis. Gastroenterology 150 (1), 194–205. 10.1053/j.gastro.2015.09.026 26416327

[B46] Yang J. Chen Y. Sun H. Zhang X. Wang J. Liang Z. (2023). Tenofovir versus entecavir on decreasing risk of HBV-related hepatocellular carcinoma recurrence after liver transplantation. Infect. agents cancer 18 (1), 2. 10.1186/s13027-022-00478-4 PMC9847063 36650583

[B47] Yip T. C. Wong V. W. Chan H. L. Tse Y. K. Lui G. C. Wong G. L. (2020). Tenofovir is associated with lower risk of hepatocellular carcinoma than entecavir in patients with chronic HBV infection in China. Gastroenterology 158 (1), 215–225. 10.1053/j.gastro.2019.09.025 31574268

[B48] Yokosuka O. Takaguchi K. Fujioka S. Shindo M. Chayama K. Kobashi H. (2010). Long-term use of entecavir in nucleoside-naïve Japanese patients with chronic hepatitis B infection. J. Hepatol. 52 (6), 791–799. 10.1016/j.jhep.2009.12.036 20409606

[B49] Yoshida K. Solomon D. H. Kim S. C. (2015). Active-comparator design and new-user design in observational studies. Nat. Rev. Rheumatol. 11 (7), 437–441. 10.1038/nrrheum.2015.30 25800216 PMC4486631

[B50] Zhang X. Guan L. Tian H. Zeng Z. Chen J. Huang D. (2021). Risk factors and prevention of viral hepatitis-related hepatocellular carcinoma. Front. Oncol. 11, 686962. 10.3389/fonc.2021.686962 34568017 PMC8458967

