# Supplementary material for: Comparative effectiveness of tenofovir versus entecavir in patients with hepatitis B virus-related cirrhosis in Taiwan: a retrospective cohort study
Source: Front Pharmacol. 2023 Dec 19;14:1301120. doi: 10.3389/fphar.2023.1301120 (PMC10763146; doi:10.3389/fphar.2023.1301120)
Supplement: Supplementary file 1 [file DataSheet1.PDF]

Supplement

eMethods 1. Definition of Previously untreated (PUT) patients and Previously treated (PT) patients.

(1) Previously untreated (PUT) patients: patients without a prescription for HBV medications during pre-cirrhosis period.

(2) Previously treated (PT) patients: patients with at least a prescription for HBV medications during pre-cirrhosis period.

eMethods 2. Definition of outcome event in details.

**Outcome**	Definition
**Primary outcome**	
**Composite outcome**	Composite outcome of HCC, LT, and all-cause mortality
**Secondary outcome**	
**Hepatocellular carcinoma**	Diagnoses of HCC, records of receiving HCC-associated procedures or HCC-related death during follow-up period.
**Liver transplantation**	Presence of diagnoses codes of LT, records of receiving LT-related procedures during follow-up period
**All-cause death**	Patients included in the Multiple cause of death data during follow-up period

HCC: Hepatocellular carcinoma, LT: Liver transplantation

Tables

eTable 1. The diagnostic codes in our study.

Comorbidity	ICD-9-CM	ICD-10-CM
**Liver disease-related comorbidities**		
**Cirrhosis**	571.2,571.3,571.5,571.6	K70.2, K70.3, K74
**Alcoholic hepatitis**	571.0	K70
**Alcoholic cirrhosis**	571.2	K70.11, K70.2, K70.3
**Biliary cirrhosis**	571.6	K74.3- K74.5
**Hepatic coma**	070.22, 070.23, 070.6, 572.2	B19.0, B19.1, K72.11, K72.9
**Ascites**	789.5	R18, K70.11, K70.31, K71.51
**Hepatorenal**	572.4	K76.7
**Hepatic**	572.2, 070.2, 070.6	B19.0,B19.1, K72.11, K72.9
**Esophageal varices with bleeding**	456.0, 456.20	I85.01, I85.11
**HBV infection**	070.2,070.3, V02.61	B18.0,B18.1,B19.1,Z22.51
**HCV infection**	070.41, 070.44, 070.54,	B17.11, B18.2, B19.2, Z22.52
**Hepatocellular**	155.0-155.2	C22.0, C22.2-C22.4, C22.7-
**Liver transplantation**	996.82, V42.7	D89.8, T86.4, Z94.4
**Non Liver disease-related comorbidities**		
**Hypertension**	401-405	I10-I15
**Hyperlipidemia**	272.0-272.5	E78.0-E78.5
**Diabetes mellitus**	250	E10-E13
**Cerebrovascular**	430-438	I60-I69, G45-G46
**Coronary artery**	410-414	I20-I25
**Chronic kidney**	585, 586, 593.9	N18, N19
**Chronic obstructive pulmonary disease**	490, 491, 492, 496	J44.0, J44.1, J44.9

ICD-9-CM: International Classification of Diseases, Ninth Revision, Clinical Modification, ICD-10-CM: International Classification of Diseases, Tenth Revision, Clinical Modification.

eTable 2. Patients' characteristics before propensity score methods.

Characteristics	PUT cohort			PT cohort		
	ETV (n=8208)	TDF/TAF (n=3663)	ASMD[a]	ETV (n=4716)	TDF/TAF (n=1764)	ASMD[a]
**Mean age (SD), y**	57.49 (11.83)	55.04 (11.71)	0.208	57.83 (11.81)	54.89 (12.40)	0.243
**Gender, n (%)**						
Male	6000 (73.1)	2709 (73.96)	0.019	3560 (75.49)	1353 (76.70)	0.028
Female	2208 (26.9)	954 (26.04)		1156 (24.51)	411 (23.30)	
**Comorbidities, n (%)**						
HCV co-infection	346 (4.22)	144 (3.93)	0.014	229 (4.86)	73 (4.14)	0.035
HDV co-infection	<3	<3	0.285	<3	<3	0.039
HEV co-infection	<3	4 (0.11)	0.039	<3	<3	0.006
HIV co-infection	8 (0.10)	20 (0.55)	0.079	4 (0.08)	20 (1.13)	0.135
Alcoholic cirrhosis	208 (2.53)	88 (2.4)	0.008	90 (1.91)	37 (2.10)	0.014
Biliary cirrhosis	<3	<3	0.008	<3	<3	0.006
Hypertension	2972 (36.21)	1122 (30.63)	0.118	1716 (36.39)	528 (29.93)	0.137
Hyperlipidemia	1524 (18.57)	630 (17.20)	0.036	800 (16.96)	311 (17.63)	0.018
Diabetes	2195 (26.74)	831 (22.69)	0.094	1261 (26.74)	384 (21.77)	0.116
Chronic kidney disease	451 (5.49)	95 (2.59)	0.148	314 (6.66)	64 (3.63)	0.138
**History of complications, n (%)**						
Ascites	321 (3.91)	106 (2.89)	0.056	196 (4.16)	78 (4.42)	0.013
Hepatic encephalophy	997 (12.15)	398 (10.87)	0.040	785 (16.65)	345 (19.56)	0.076
EVB	116 (1.41)	51 (1.39)	0.002	82 (1.74)	18 (1.02)	0.062
Hepatorenal syndrome	14 (0.17)	<3	0.035	8 (0.17)	5 (0.28)	0.024
**Charlson Comorbidity Index**						
Mean (SD)	1.68 (1.80)	1.38 (1.58)	0.087	2.36 (2.17)	2.15 (2.06)	0.140
**Disease progression period (y)**	2.23 (2.23)	2.46 (2.33)	0.101	2.90 (2.32)	3.66 (2.37)	0.322
**Treatment gap period (y)**	1.01 (1.69)	1.06 (1.72)	0.027	0.17 (0.61)	0.21 (0.76)	0.069

eTable 2. Patients' characteristics before propensity score methods.

(continued.)

**Characteristics**	**PUT cohort**			**PT cohort**		
	**ETV (n=8208)**	**TDF/TAF (n=3663)**	**ASMD[a]**	**ETV (n=4716)**	**TDF/TAF (n=1764)**	**ASMD[a]**
**Co-medications, n (%)**						
ACEIs/ARBs	1839 (22.40)	663 (18.1)	0.107	1146 (24.30)	312 (17.69)	0.163
β -blockers	1162 (14.16)	471 (12.86)	0.038	838 (17.77)	233 (13.21)	0.126
Non selective	523 (6.37)	218 (5.95)	0.017	419 (8.88)	116 (6.58)	0.087
Selective	702 (8.55)	271 (7.40)	0.043	476 (10.09)	128 (7.26)	0.101
CCBs	1568 (19.10)	587 (16.03)	0.081	996 (21.12)	262 (14.85)	0.164
Diuretics	1330 (16.20)	436 (11.90)	0.124	1028 (21.80)	276 (15.65)	0.158
Furosemide	427 (5.20)	100 (2.73)	0.127	411 (8.72)	94 (5.33)	0.133
Spironolactone	250 (3.05)	69 (1.88)	0.075	355 (7.53)	98 (5.56)	0.080
Insulin	347 (4.23)	110 (3.00)	0.066	246 (5.22)	71 (4.02)	0.057
Biguanide	1318 (16.06)	526 (14.36)	0.047	800 (16.96)	267 (15.14)	0.050
Meglitinide	159 (1.94)	42 (1.15)	0.064	113 (2.40)	26 (1.47)	0.067
Sulfonylurea	1052 (12.82)	377 (10.29)	0.079	583 (12.36)	191 (10.83)	0.048
α-glucosidase inhibitors	251 (3.06)	101 (2.76)	0.018	144 (3.05)	43 (2.44)	0.038
Thiazolidinediones	230 (2.80)	76 (2.07)	0.047	101 (2.14)	39 (2.21)	0.005
DPP-4 inhibitors	664 (8.09)	237 (6.47)	0.062	416 (8.82)	149 (8.45)	0.013
SGLT2 inhibitors	43 (0.52)	34 (0.93)	0.048	37 (0.78)	14 (0.79)	0.001
GLP1 agonists	10 (0.12)	5 (0.14)	0.004	9 (0.19)	3 (0.17)	0.005
Statin	909 (11.07)	358 (9.77)	0.043	599 (12.70)	190 (10.77)	0.060
Fibrates	182 (2.22)	79 (2.16)	0.004	105 (2.23)	33 (1.87)	0.025
Silymarin	1955 (23.82)	886 (24.19)	0.009	898 (19.04)	387 (21.94)	0.072

Abbreviations: ASMD, absolute standardized mean difference; ETV, Entecavir; TDF/TAF, Tenofovir Disoproxil Fumarate/ Tenofovir Alafenamide Fumarate; HCV, Hepatitis C virus; HDV, Hepatitis D virus; HIV, Hepatitis I virus; EVB, Esophageal varices with bleeding; ACEIs, Angiotensin-converting enzyme inhibitors; ARBs, Angiotensin II receptor blockers; CCBs, Calcium-channel blockers; SGLT2, Sodium-glucose cotransporter-2; GLP1, Glucagon-like peptide-1; y, year.

[a] The absolute standardized mean difference less than 0.1 indicates well-balanced between groups.

eTable 3. Patients' characteristics within PT cohort after propensity score methods.

Characteristics	Population after PSM			Population after IPTW		
	ETV (n = 1762)	TDF/TAF (n = 1762)	ASMD[a]	ETV (n = 4717)	TDF/TAF (n = 1764)	ASMD[a]
**Mean age (SD), y**	55.05 (11.76)	54.88 (12.40)	0.013	57.06 (11.87)	57.33 (12.56)	0.022
**Gender, n (%)**						
Male	1343 (76.22)	1351 (76.67)	0.011	3578 (75.85)	1341 (76.03)	0.004
Female	419 (23.78)	411 (23.33)		1139 (24.15)	423 (23.97)	
**Comorbidities, n (%)**						
HCV co-infection	76 (4.31)	73 (4.14)	0.008	221 (4.68)	87 (4.92)	0.011
HDV co-infection	<3	<3	0.000	3 (0.07)	<3	0.002
HEV co-infection	<3	<3	0.019	<3	<3	0.006
HIV co-infection	3 (0.17)	20 (1.14)	0.120	5 (0.1)	21 (1.20)	0.137
Alcoholic cirrhosis	38 (2.16)	37 (2.10)	0.004	92 (1.96)	35 (1.98)	0.001
Biliary cirrhosis	<3	<3	0.000	<3	<3	0.005
Hypertension	527 (29.91)	528 (29.97)	0.001	1638 (34.74)	621 (35.23)	0.010
Hyperlipidemia	289 (16.40)	311 (17.65)	0.033	791 (16.77)	321 (18.17)	0.037
Diabetes	371 (21.06)	384 (21.79)	0.018	1200 (25.45)	453 (25.69)	0.006
Chronic kidney disease	63 (3.58)	64 (3.63)	0.003	275 (5.84)	102 (5.77)	0.003
**History of complications, n (%)**						
Ascites	72 (4.09)	78 (4.43)	0.017	198 (4.20)	72 (4.06)	0.007
Hepatic encephalophy	365 (20.72)	345 (19.58)	0.028	832 (17.64)	324 (18.37)	0.019
EVB	28 (1.59)	18 (1.02)	0.050	73 (1.55)	27 (1.54)	0.001
Hepatorenal syndrome	4 (0.23)	5 (0.28)	0.011	9 (0.19)	3 (0.17)	0.003
**Charlson Comorbidity Index**						
Mean (SD)	2.14 (2.02)	2.15 (2.06)	0.066	2.31 (2.14)	2.34 (2.17)	0.044
**Disease progression period (y)**	3.67 (2.51)	3.65 (2.37)	0.006	3.11 (2.41)	3.14 (2.30)	0.011
**Treatment gap period (y)**	0.22 (0.77)	0.21 (0.76)	0.011	0.18 (0.65)	0.18 (0.67)	0.001

eTable 3. Patients' characteristics within PT cohort after propensity score methods. (continued.)

Characteristics	Population after PSM			Population after IPTW		
	ETV	TDF/TAF	ASMD[a]	ETV	TDF/TAF	ASMD[a]
	(n = 1762)	(n = 1762)		(n = 4717)	(n = 1764)	
**Co-medications, n (%)**						
ACEIs/ARBs	372 (21.11)	312 (17.71)	0.086	1098 (23.28)	358 (20.28)	0.073
β -blockers	274 (15.55)	233 (13.22)	0.066	803 (17.01)	269 (15.23)	0.048
Non selective	133 (7.55)	116 (6.58)	0.038	400 (8.49)	135 (7.68)	0.030
Selective	156 (8.85)	128 (7.26)	0.058	454 (9.63)	146 (8.28)	0.047
CCBs	323 (18.33)	262 (14.87)	0.093	948 (20.1)	306 (17.33)	0.071
Diuretics	345 (19.58)	276 (15.66)	0.103	983 (20.84)	328 (18.61)	0.056
Furosemide	139 (7.89)	94 (5.33)	0.103	389 (8.24)	121 (6.86)	0.052
Spironolactone	125 (7.09)	98 (5.56)	0.063	340 (7.20)	124 (7.04)	0.006
Insulin	69 (3.92)	71 (4.03)	0.006	234 (4.95)	80 (4.56)	0.019
Biguanide	266 (15.1)	267 (15.15)	0.002	775 (16.43)	302 (17.10)	0.018
Meglitinide	27 (1.53)	26 (1.48)	0.005	106 (2.24)	36 (2.02)	0.015
Sulfonylurea	176 (9.99)	191 (10.84)	0.028	555 (11.76)	229 (13.00)	0.037
α-glucosidase inhibitors	42 (2.38)	43 (2.44)	0.004	138 (2.92)	52 (2.95)	0.001
Thiazolidinediones	39 (2.21)	39 (2.21)	0.000	100 (2.11)	43 (2.42)	0.021
DPP-4 inhibitors	141 (8.00)	149 (8.46)	0.017	402 (8.51)	176 (9.97)	0.050
SGLT2 inhibitors	16 (0.91)	14 (0.79)	0.012	40 (0.85)	14 (0.82)	0.003
GLP1 agonists	4 (0.23)	3 (0.17)	0.013	9 (0.20)	3 (0.17)	0.010
Statin	218 (12.37)	190 (10.78)	0.050	586 (12.42)	195 (11.04)	0.043
Fibrates	37 (2.10)	33 (1.87)	0.016	104 (2.20)	31 (1.76)	0.031
Silymarin	332 (18.84)	387 (21.96)	0.078	898 (19.03)	392 (22.20)	0.078

Abbreviations: IPTW, inverse probability of treatment weighting; ASMD, absolute standardized mean difference; ETV, Entecavir; TDF/TAF, Tenofovir Disoproxil Fumarate/ Tenofovir Alafenamide Fumarate; HCV, Hepatitis C virus; HDV, Hepatitis D virus; HIV, Hepatitis I virus; EVB, Esophageal varices with bleeding; ACEIs, Angiotensin-converting enzyme inhibitors; ARBs, Angiotensin II receptor blockers; CCBs, Calcium-channel blockers; SGLT2, Sodium-glucose cotransporter-2; GLP1, Glucagon-like peptide-1; y, year.

[a] The absolute standardized mean difference less than 0.1 indicates well-balanced between groups.

eTable 4. Patients' characteristics within PUT cohort after propensity score methods for the composite outcome analysis.

Characteristics	Population after PSM			Population after IPTW		
	ETV (n = 3417)	TDF/TAF (n = 3417)	ASMD[a]	ETV (n = 7464)	TDF/TAF (n = 3420)	ASMD[a]
**Mean age (SD), y**	54.84 (11.92)	54.71 (11.73)	0.010	56.48 (11.91)	56.42 (11.76)	0.005
**Gender, n (%)**						
Male	2522 (73.81)	2521 (73.78)	0.001	5446 (72.97)	2498 (73.03)	0.001
Female	895 (26.19)	896 (26.22)	0.001	2018 (27.03)	922 (26.97)	0.001
**Comorbidities, n (%)**						
HCV co-infection	126 (3.69)	126 (3.69)	0.000	301 (4.03)	137 (4.00)	0.001
HDV co-infection	<3	<3	0.000	<3	<3	0.288
HEV co-infection	<3	<3	0.000	<3	<3	0.018
HIV co-infection	<3	20 (0.59)	0.093	7.72 (0.1)	22 (0.63)	0.087
Alcoholic cirrhosis	88 (2.58)	86 (2.52)	0.004	194 (2.6)	88 (2.58)	0.000
Biliary cirrhosis	<3	<3	0.004	<3	<3	0.000
Hypertension	1054 (30.85)	1036 (30.32)	0.011	2567 (34.39)	1171 (34.24)	0.003
Hyperlipidemia	610 (17.85)	599 (17.53)	0.008	1358 (18.2)	636 (18.6)	0.010
Diabetes	756 (22.12)	759 (22.21)	0.002	1888 (25.29)	866 (25.33)	0.001
Chronic kidney disease	80 (2.34)	88 (2.58)	0.015	350 (4.69)	157 (4.58)	0.005
**History of complications, n (%)**						
Ascites	104 (3.04)	103 (3.01)	0.002	282 (3.78)	126 (3.67)	0.006
Hepatic encephalophy	376 (11.00)	378 (11.06)	0.002	904 (12.11)	419 (12.25)	0.004
EVB	51 (1.49)	50 (1.46)	0.002	108 (1.44)	50 (1.47)	0.002
Hepatorenal syndrome	<3	<3	0.164	10 (0.14)	4 (0.12)	0.006
**Charlson Comorbidity Index**						
Mean (SD)	1.36 (1.54)	1.38 (1.56)	0.034	1.59 (1.74)	1.59 (1.76)	0.041
**Disease progression period (y)**	2.46 (2.33)	2.49 (2.35)	0.011	2.34 (2.28)	2.33 (2.29)	0.001
**Treatment gap period (y)**	0.98 (1.73)	0.95 (1.63)	0.018	0.90 (1.61)	0.91 (1.58)	0.004

eTable 4. Patients' characteristics within PUT cohort after propensity score methods for the composite outcome analysis. (continued.)

Characteristics	Population after PSM			Population after IPTW		
	ETV (n = 3417)	TDF/TAF (n = 3417)	ASMD[a]	ETV (n = 7464)	TDF/TAF (n = 3420)	ASMD[a]
**Co-medications, n (%)**						
ACEIs/ARBs	629 (18.41)	613 (17.94)	0.012	1591 (21.32)	704 (20.58)	0.018
β -blockers	400 (11.71)	444 (12.99)	0.039	1005 (13.47)	506 (14.78)	0.038
Non selective	182 (5.33)	210 (6.15)	0.035	452 (6.05)	238 (6.97)	0.037
Selective	239 (6.99)	252 (7.37)	0.015	608 (8.14)	293 (8.58)	0.016
CCBs	547 (16.01)	542 (15.86)	0.004	1330 (17.82)	626 (18.31)	0.013
Diuretics	453 (13.26)	401 (11.74)	0.046	1140 (15.27)	476 (13.92)	0.038
Furosemide	135 (3.95)	94 (2.75)	0.067	367 (4.92)	123 (3.61)	0.065
Spironolactone	81 (2.37)	62 (1.81)	0.039	221 (2.96)	75 (2.18)	0.050
Insulin	120 (3.51)	101 (2.96)	0.031	295 (3.95)	122 (3.56)	0.021
Biguanide	470 (13.75)	480 (14.05)	0.008	1154 (15.46)	533 (15.59)	0.004
Meglitinide	41 (1.20)	38 (1.11)	0.008	133 (1.79)	47 (1.37)	0.033
Sulfonylurea	376 (11.00)	342 (10.01)	0.032	909 (12.18)	386 (11.29)	0.028
α-glucosidase inhibitors	80 (2.34)	91 (2.66)	0.021	213 (2.85)	105 (3.06)	0.012
Thiazolidinediones	76 (2.22)	70 (2.05)	0.012	204 (2.74)	82 (2.4)	0.021
DPP-4 inhibitors	219 (6.41)	228 (6.67)	0.011	590 (7.91)	260 (7.6)	0.012
SGLT2 inhibitors	15 (0.44)	34 (1.00)	0.066	40 (0.53)	36 (1.04)	0.058
GLP1 agonists	4 (0.12)	5 (0.15)	0.008	10 (0.13)	5 (0.14)	0.004
Statin	326 (9.54)	341 (9.98)	0.015	796 (10.66)	381 (11.15)	0.015
Fibrates	71 (2.08)	77 (2.25)	0.012	171 (2.29)	78 (2.29)	0.000
Silymarin	796 (23.30)	829 (24.26)	0.023	1776 (23.79)	859 (25.1)	0.030

Abbreviations: IPTW, inverse probability of treatment weighting; ASMD, absolute standardized mean difference; ETV, Entecavir; TDF/TAF, Tenofovir Disoproxil Fumarate/ Tenofovir Alafenamide Fumarate; HCV, Hepatitis C virus; HDV, Hepatitis D virus; HIV, Hepatitis I virus; EVB, Esophageal varices with bleeding; ACEIs, Angiotensin-converting enzyme inhibitors; ARBs, Angiotensin II receptor blockers; CCBs, Calcium-channel blockers; SGLT2, Sodium-glucose cotransporter-2; GLP1, Glucagon-like peptide-1; y, year.

[a] The absolute standardized mean difference less than 0.1 indicates well-balanced between groups.

eTable 5. Patients' characteristics within PT cohort after propensity score methods for the composite outcome analysis.

Characteristics	Population after PSM			Population after IPTW		
	ETV (n=1724)	TDF/TAF (n=1724)	ASMD[a]	ETV (n=4611)	TDF/TAF (n=1727)	ASMD[a]
**Mean age (SD), y**	55.21 (11.89)	54.89 (12.45)	0.027	56.48 (11.91)	56.42 (11.76)	0.022
**Gender, n (%)**						
Male	1316 (76.33)	1318 (76.45)	0.003	5446 (72.97)	2498 (73.03)	0.003
Female	408 (23.67)	406 (23.55)	0.003	2018 (27.03)	922 (26.97)	0.003
**Comorbidities, n (%)**						
HCV co-infection	66 (3.83)	70 (4.06)	0.012	301 (4.03)	137 (4.00)	0.013
HDV co-infection	<3	<3	0.000	<3	<3	0.002
HEV co-infection	<3	<3	0.020	<3	<3	0.007
HIV co-infection	<3	19 (1.10)	0.117	7.72 (0.1)	21.58 (0.63)	0.135
Alcoholic cirrhosis	36 (2.09)	37 (2.15)	0.004	194 (2.6)	88 (2.58)	0.001
Biliary cirrhosis	<3	<3	0.198	<3	<3	0.005
Hypertension	492 (28.54)	516 (29.93)	0.031	2567 (34.39)	1171 (34.24)	0.010
Hyperlipidemia	280 (16.24)	301 (17.46)	0.033	1358 (18.2)	636 (18.60)	0.034
Diabetes	360 (20.88)	372 (21.58)	0.017	1888 (25.29)	866 (25.33)	0.007
Chronic kidney disease	57 (3.31)	62 (3.60)	0.016	350 (4.69)	157 (4.58)	0.003
**History of complications, n (%)**						
Ascites	67 (3.89)	77 (4.47)	0.029	282 (3.78)	126 (3.67)	0.007
Hepatic encephalophy	324 (18.79)	329 (19.08)	0.007	904 (12.11)	419 (12.25)	0.019
EVB	15 (0.87)	17 (0.99)	0.012	108 (1.44)	50 (1.47)	0.001
Hepatorenal syndrome	<3	<3	0.015	10 (0.14)	4 (0.12)	0.003
**Charlson Comorbidity Index**			0.051			0.040
Mean (SD)	2.16 (2.04)	2.14 (2.06)		1.59 (1.74)	1.59 (1.76)	
**Disease progression period (y)**	3.66 (2.53)	3.64 (2.36)	0.008	3.11 (2.41)	3.14 (2.29)	0.012
**Treatment gap period (y)**	0.20 (0.75)	0.19 (0.73)	0.010	0.15 (0.57)	0.15 (0.60)	0.000

eTable 5. Patients' characteristics within PT cohort after propensity score methods for the composite outcome analysis (continued.)

Characteristics	Population after PSM			Population after IPTW		
	ETV (n=1724)	TDF/TAF (n=1724)	ASMD[a]	ETV (n=4611)	TDF/TAF (n=1727)	ASMD[a]
**Co-medications, n (%)**						
ACEIs/ARBs	342 (19.84)	306 (17.75)	0.054	1591 (21.32)	704 (20.58)	0.072
β -blockers	258 (14.97)	230 (13.34)	0.047	1005 (13.47)	506 (14.78)	0.044
Non selective	122 (7.08)	115 (6.67)	0.016	452 (6.05)	238 (6.97)	0.025
Selective	148 (8.58)	126 (7.31)	0.047	608 (8.14)	293 (8.58)	0.044
CCBs	298 (17.29)	257 (14.91)	0.065	1330 (17.82)	626 (18.31)	0.068
Diuretics	317 (18.39)	269 (15.60)	0.074	1140 (15.27)	476 (13.92)	0.056
Furosemide	128 (7.42)	92 (5.34)	0.086	367 (4.92)	123 (3.61)	0.050
Spironolactone	105 (6.09)	96 (5.57)	0.022	221 (2.96)	75 (2.18)	0.004
Insulin	61 (3.54)	69 (4.00)	0.024	295 (3.95)	122 (3.56)	0.015
Biguanide	248 (14.39)	261 (15.14)	0.021	1154 (15.46)	533 (15.59)	0.023
Meglitinide	30 (1.74)	26 (1.51)	0.018	133 (1.79)	47 (1.37)	0.014
Sulfonylurea	159 (9.22)	187 (10.85)	0.054	909 (12.18)	386 (11.29)	0.041
α-glucosidase inhibitors	47 (2.73)	43 (2.49)	0.015	213 (2.85)	105 (3.06)	0.006
Thiazolidinediones	36 (2.09)	37 (2.15)	0.004	204 (2.74)	82 (2.4)	0.016
DPP-4 inhibitors	125 (7.25)	145 (8.41)	0.043	590 (7.91)	260 (7.6)	0.050
SGLT2 inhibitors	17 (0.99)	13 (0.75)	0.025	40 (0.53)	36 (1.04)	0.006
GLP1 agonists	<3	<3	0.020	10 (0.13)	5 (0.14)	0.048
Statin	190 (11.02)	186 (10.79)	0.007	796 (10.66)	381 (11.15)	0.039
Fibrates	33 (1.91)	30 (1.74)	0.013	171 (2.29)	78 (2.29)	0.038
Silymarin	332 (19.26)	377 (21.87)	0.065	1776 (23.79)	859 (25.1)	0.079

Abbreviations: IPTW, inverse probability of treatment weighting; ASMD, absolute standardized mean difference; ETV, Entecavir; TDF/TAF, Tenofovir Disoproxil Fumarate/ Tenofovir Alafenamide Fumarate; HCV, Hepatitis C virus; HDV, Hepatitis D virus; HIV, Hepatitis I virus; EVB, Esophageal varices with bleeding; ACEIs, Angiotensin-converting enzyme inhibitors; ARBs, Angiotensin II receptor blockers; CCBs, Calcium-channel blockers; SGLT2, Sodium-glucose cotransporter-2; GLP1, Glucagon-like peptide-1; y, year.

[a] The absolute standardized mean difference less than 0.1 indicates well-balanced between groups.

eTable 6. Patients' characteristics within PUT cohort after propensity score methods for the HCC analysis.

Characteristics	Population after PSM			Population after IPTW		
	ETV (n = 3423)	TDF/TAF (n = 3423)	ASMD[a]	ETV (n = 7496)	TDF/TAF (n = 3427)	ASMD[a]
**Mean age (SD), y**	54.88 (11.94)	54.71 (11.73)	0.014	56.48 (11.88)	56.43 (11.81)	0.004
**Gender, n (%)**						
Male	2536 (74.09)	2526 (73.79)	0.007	5469 (72.99)	2503 (73.07)	0.002
Female	887 (25.91)	897 (26.21)	0.007	2024 (27.01)	922 (26.93)	0.002
**Comorbidities, n (%)**						
HCV co-infection	126 (3.68)	126 (3.68)	0.000	302 (4.03)	137 (4.01)	0.001
HDV co-infection	<3	<3	0.000	<3	<3	0.288
HEV co-infection	<3	<3	0.000	<3	<3	0.018
HIV co-infection	5 (0.15)	20 (0.58)	0.073	8 (0.10)	22 (0.63)	0.088
Alcoholic cirrhosis	83 (2.42)	86 (2.51)	0.006	194 (2.59)	88 (2.57)	0.001
Biliary cirrhosis	<3	<3	0.006	<3	<3	0.001
Hypertension	1065 (31.11)	1036 (30.27)	0.018	2575 (34.37)	1172 (34.23)	0.003
Hyperlipidemia	595 (17.38)	599 (17.5)	0.003	1368 (18.25)	636 (18.56)	0.008
Diabetes	761 (22.23)	761 (22.23)	0.000	1898 (25.33)	870 (25.41)	0.002
Chronic kidney disease	81 (2.37)	88 (2.57)	0.013	354 (4.72)	158 (4.60)	0.006
**History of complications, n (%)**						
Ascites	92 (2.69)	103 (3.01)	0.019	285 (3.80)	127 (3.70)	0.005
Hepatic encephalophy	428 (12.5)	379 (11.07)	0.044	911 (12.16)	421 (12.3)	0.004
EVB	57 (1.67)	50 (1.46)	0.016	108 (1.45)	51 (1.48)	0.003
Hepatorenal syndrome	<3	<3	0.014	11 (0.15)	4.35 (0.13)	0.005
**Charlson Comorbidity Index**						
Mean (SD)	1.39 (1.52)	1.38 (1.56)	0.030	1.60 (1.74)	1.74 (1.77)	0.041
**Disease progression period (y)**	2.54 (2.36)	2.49 (2.35)	0.023	2.34 (2.28)	2.34 (2.29)	0.001
**Treatment gap period (y)**	0.94 (1.65)	0.95 (1.63)	0.008	0.90 (1.61)	0.91 (1.58)	0.004

eTable 6. Patients' characteristics within PUT cohort after propensity score methods for the HCC analysis. (continued.)

Characteristics	Population after PSM			Population after IPTW		
	ETV (n = 3423)	TDF/TAF (n = 3423)	ASMD[a]	ETV (n = 7496)	TDF/TAF (n = 3427)	ASMD[a]
**Co-medications, n (%)**						
ACEIs/ARBs	648 (18.93)	613 (17.91)	0.026	1598 (21.32)	705 (20.58)	0.018
β -blockers	440 (12.85)	444 (12.97)	0.003	1011 (13.49)	506 (14.77)	0.037
Non selective	199 (5.81)	210 (6.13)	0.014	453 (6.05)	239 (6.97)	0.037
Selective	263 (7.68)	252 (7.36)	0.012	612 (8.16)	294 (8.58)	0.015
CCBs	532 (15.54)	542 (15.83)	0.008	1336 (17.83)	627 (18.31)	0.012
Diuretics	441 (12.88)	402 (11.74)	0.035	1147 (15.31)	478 (13.96)	0.038
Furosemide	136 (3.97)	95 (2.78)	0.066	369 (4.92)	125 (3.66)	0.062
Spironolactone	87 (2.54)	63 (1.84)	0.048	223 (2.98)	76 (2.22)	0.048
Insulin	118 (3.45)	101 (2.95)	0.028	295 (3.94)	122 (3.56)	0.020
Biguanide	486 (14.20)	481 (14.05)	0.004	1159 (15.47)	535 (15.63)	0.004
Meglitinide	41 (1.20)	38 (1.11)	0.008	133 (1.78)	47 (1.37)	0.032
Sulfonylurea	377 (11.01)	342 (9.99)	0.033	913 (12.19)	387 (11.29)	0.028
α-glucosidase inhibitors	78 (2.28)	91 (2.66)	0.024	213 (2.84)	105 (3.06)	0.013
Thiazolidinediones	87 (2.54)	70 (2.04)	0.033	205 (2.74)	82 (2.4)	0.021
DPP-4 inhibitors	226 (6.60)	228 (6.66)	0.002	593 (7.92)	260 (7.59)	0.012
SGLT2 inhibitors	18 (0.53)	34 (0.99)	0.054	40 (0.53)	36 (1.04)	0.058
GLP1 agonists	<3	5 (0.15)	0.017	10 (0.13)	5 (0.14)	0.004
Statin	311 (9.09)	341 (9.96)	0.030	802 (10.71)	381 (11.13)	0.014
Fibrates	73 (2.13)	77 (2.25)	0.008	171 (2.29)	78 (2.27)	0.001
Silymarin	812 (23.72)	829 (24.22)	0.012	1782 (23.78)	858 (25.06)	0.030

Abbreviations: IPTW, inverse probability of treatment weighting; ASMD, absolute standardized mean difference; ETV, Entecavir; TDF/TAF, Tenofovir Disoproxil Fumarate/ Tenofovir Alafenamide Fumarate; HCV, Hepatitis C virus; HDV, Hepatitis D virus; HIV, Hepatitis I virus; EVB, Esophageal varices with bleeding; ACEIs, Angiotensin-converting enzyme inhibitors; ARBs, Angiotensin II receptor blockers; CCBs, Calcium-channel blockers; SGLT2, Sodium-glucose cotransporter-2; GLP1, Glucagon-like peptide-1; y, year.

[a] The absolute standardized mean difference less than 0.1 indicates well-balanced between groups.

eTable 7. Patients' characteristics within PT cohort after propensity score methods for the HCC analysis.

Characteristics	Population after PSM			Population after IPTW		
	ETV (n = 1737)	TDF/TAF (n = 1737)	ASMD[a]	ETV (n = 4633)	TDF/TAF (n = 1738)	ASMD[a]
**Mean age (SD), y**	55.21 (11.87)	54.87 (12.44)	0.028	57.01 (11.88)	57.27 (12.58)	0.021
**Gender, n (%)**						
Male	1347 (77.55)	1331 (76.63)	0.022	3509 (75.74)	1318 (75.86)	0.003
Female	390 (22.45)	406 (23.37)	0.022	1124 (24.26)	420 (24.14)	0.003
**Comorbidities, n (%)**						
HCV co-infection	75 (4.32)	71 (4.09)	0.011	214 (4.63)	85 (4.88)	0.012
HDV co-infection	<3	<3	0.000	3 (0.07)	<3	0.002
HEV co-infection	<3	<3	0.020	<3	<3	0.006
HIV co-infection	3 (0.17)	19 (1.09)	0.116	5 (0.11)	21 (1.18)	0.135
Alcoholic cirrhosis	40 (2.3)	37 (2.13)	0.012	90 (1.94)	34 (1.96)	0.001
Biliary cirrhosis	<3	<3	0.000	<3	<3	0.005
Hypertension	527 (30.34)	522 (30.05)	0.006	1602 (34.58)	610 (35.09)	0.011
Hyperlipidemia	295 (16.98)	303 (17.44)	0.012	778 (16.8)	314 (18.04)	0.033
Diabetes	365 (21.01)	376 (21.65)	0.015	1174 (25.35)	447 (25.7)	0.008
Chronic kidney disease	59 (3.4)	63 (3.63)	0.013	268 (5.78)	99 (5.71)	0.003
**History of complications, n (%)**						
Ascites	79 (4.55)	78 (4.49)	0.003	198 (4.27)	72 (4.12)	0.007
Hepatic encephalophy	331 (19.06)	336 (19.34)	0.007	815 (17.59)	319 (18.33)	0.019
EVB	21 (1.21)	18 (1.04)	0.016	72 (1.55)	27 (1.54)	0.001
Hepatorenal syndrome	<3	5 (0.29)	0.024	9 (0.19)	3.05 (0.18)	0.004
**Charlson Comorbidity Index**						
Mean (SD)	2.16 (2.12)	2.15 (2.06)	0.046	2.31 (2.15)	2.35 (2.18)	0.042
**Disease progression period (y)**	3.66 (2.55)	3.65 (2.37)	0.005	3.11 (2.41)	3.13 (2.29)	0.012
**Treatment gap period (y)**	0.20 (0.70)	0.19 (0.73)	0.006	1.85 (6.94)	0.15 (0.61)	0.001

eTable 7. Patients' characteristics within PT cohort after propensity score methods for the HCC analysis. (continued.)

Characteristics	Population after PSM			Population after IPTW		
	ETV (n = 1737)	TDF/TAF (n = 1737)	ASMD[a]	ETV (n = 4633)	TDF/TAF (n = 1738)	ASMD[a]
**Co-medications, n (%)**						
ACEIs/ARBs	361 (20.78)	308 (17.73)	0.077	1080 (23.32)	352 (20.23)	0.075
β -blockers	250 (14.39)	232 (13.36)	0.030	783 (16.90)	266 (15.3)	0.044
Non selective	110 (6.33)	115 (6.62)	0.012	391 (8.44)	134 (7.71)	0.027
Selective	149 (8.58)	128 (7.37)	0.045	444 (9.58)	145 (8.33)	0.044
CCBs	294 (16.93)	260 (14.97)	0.053	925 (19.96)	301 (17.34)	0.067
Diuretics	328 (18.88)	273 (15.72)	0.084	966 (20.86)	323 (18.6)	0.057
Furosemide	129 (7.43)	94 (5.41)	0.082	383 (8.26)	121 (6.94)	0.050
Spironolactone	125 (7.2)	97 (5.58)	0.066	335 (7.23)	123 (7.07)	0.006
Insulin	63 (3.63)	69 (3.97)	0.018	227 (4.9)	79 (4.55)	0.017
Biguanide	266 (15.31)	261 (15.03)	0.008	758 (16.36)	296 (17.06)	0.019
Meglitinide	33 (1.9)	26 (1.5)	0.031	105 (2.26)	36 (2.04)	0.015
Sulfonylurea	177 (10.19)	187 (10.77)	0.019	544 (11.74)	226 (12.99)	0.038
α-glucosidase inhibitors	44 (2.53)	43 (2.48)	0.004	135 (2.92)	52 (2.98)	0.004
Thiazolidinediones	44 (2.53)	37 (2.13)	0.027	100 (2.15)	41 (2.34)	0.012
DPP-4 inhibitors	137 (7.89)	145 (8.35)	0.017	395 (8.53)	172 (9.92)	0.048
SGLT2 inhibitors	17 (0.98)	13 (0.75)	0.025	39 (0.84)	14 (0.78)	0.006
GLP1 agonists	3 (0.17)	<3	0.034	9 (0.2)	<3	0.048
Statin	212 (12.2)	187 (10.77)	0.045	574 (12.39)	192 (11.06)	0.041
Fibrates	36 (2.07)	31 (1.78)	0.021	102 (2.20)	29 (1.68)	0.038
Silymarin	351 (20.21)	380 (21.88)	0.041	881 (19.01)	385 (22.14)	0.077

Abbreviations: IPTW, inverse probability of treatment weighting; ASMD, absolute standardized mean difference; ETV, Entecavir; TDF/TAF, Tenofovir Disoproxil Fumarate/ Tenofovir Alafenamide Fumarate; HCV, Hepatitis C virus; HDV, Hepatitis D virus; HIV, Hepatitis I virus; EVB, Esophageal varices with bleeding; ACEIs, Angiotensin-converting enzyme inhibitors; ARBs, Angiotensin II receptor blockers; CCBs, Calcium-channel blockers; SGLT2, Sodium-glucose cotransporter-2; GLP1, Glucagon-like peptide-1; y, year.

[a] The absolute standardized mean difference less than 0.1 indicates well-balanced between groups.

eTable 8. Patients' characteristics within PUT cohort after propensity score methods for liver transplantation analysis.

Characteristics	Population after PSM			Population after IPTW		
	ETV (n = 3651)	TDF/TAF (n = 3651)	ASMD[a]	ETV (n = 8160)	TDF/TAF (n = 3655)	ASMD[a]
**Mean age (SD), y**	55.18 (11.83)	55.04 (11.70)	0.012	56.75 (11.84)	56.71 (11.78)	0.003
**Gender, n (%)**						
Male	2724 (74.61)	2699 (73.92)	0.016	5984 (73.33)	2682 (73.38)	0.001
Female	927 (25.39)	952 (26.08)	0.016	2177 (26.67)	973 (26.62)	0.001
**Comorbidities, n (%)**						
HCV co-infection	145 (3.97)	144 (3.94)	0.001	335 (4.10)	148 (4.05)	0.002
HDV co-infection	<3	<3	0.001	<3	<3	0.290
HEV co-infection	<3	<3	0.001	<3	<3	0.017
HIV co-infection	5 (0.14)	20 (0.55)	0.070	8 (0.10)	21 (0.58)	0.084
Alcoholic cirrhosis	82 (2.25)	88 (2.41)	0.011	204 (2.50)	91 (2.50)	0.000
Biliary cirrhosis	<3	<3	0.011	<3	<3	0.000
Hypertension	1130 (30.95)	1120 (30.68)	0.006	2820 (34.55)	1262 (34.52)	0.001
Hyperlipidemia	626 (17.15)	629 (17.23)	0.002	1483 (18.18)	663 (18.13)	0.001
Diabetes	852 (23.34)	827 (22.65)	0.016	2080 (25.49)	935 (25.59)	0.002
Chronic kidney disease	97 (2.66)	95 (2.60)	0.003	373 (4.58)	164 (4.49)	0.004
**History of complications, n (%)**						
Ascites	108 (2.96)	106 (2.9)	0.003	291 (3.57)	127 (3.48)	0.005
Hepatic encephalophy	431 (11.8)	395 (10.82)	0.031	956 (11.72)	432 (11.82)	0.003
EVB	45 (1.23)	51 (1.40)	0.014	115 (1.41)	53 (1.44)	0.003
Hepatorenal syndrome	<3	<3	0.014	10 (0.13)	4 (0.10)	0.007
**Charlson Comorbidity Index**						
Mean (SD)	1.39 (1.53)	1.38 (1.57)	0.048	1.58 (1.73)	1.59 (1.77)	0.039
**Disease progression period (y)**	2.52 (2.36)	2.46 (2.33)	0.023	2.30 (2.27)	2.30 (2.27)	0.002
**Treatment gap period (y)**	1.06 (1.76)	1.05 (1.71)	0.010	1.02 (1.71)	1.02 (1.69)	0.004

eTable 8. Patients' characteristics within PUT cohort after propensity score methods for liver transplantation analysis. (continued.)

Characteristics	Population after PSM			Population after IPTW		
	ETV (n = 3651)	TDF/TAF (n = 3651)	ASMD[a]	ETV (n = 8160)	TDF/TAF (n = 3655)	ASMD[a]
**Co-medications, n (%)**						
ACEIs/ARBs	707 (19.36)	661 (18.10)	0.032	1739 (21.31)	752 (20.57)	0.018
β -blockers	444 (12.16)	471 (12.90)	0.022	1104 (13.52)	531 (14.53)	0.029
Non selective	195 (5.34)	218 (5.97)	0.027	498 (6.10)	245 (6.71)	0.025
Selective	270 (7.40)	271 (7.42)	0.001	665 (8.15)	311 (8.52)	0.013
CCBs	571 (15.64)	585 (16.02)	0.011	1473 (18.05)	672 (18.40)	0.009
Diuretics	484 (13.26)	433 (11.86)	0.042	1245 (15.26)	509 (13.93)	0.037
Furosemide	138 (3.78)	99 (2.71)	0.060	390 (4.78)	129 (3.54)	0.062
Spironolactone	91 (2.49)	68 (1.86)	0.043	233 (2.85)	82 (2.24)	0.039
Insulin	128 (3.51)	110 (3.01)	0.028	324 (3.97)	130 (3.56)	0.022
Biguanide	531 (14.54)	524 (14.35)	0.005	1262 (15.46)	577 (15.78)	0.009
Meglitinide	45 (1.23)	42 (1.15)	0.008	144 (1.77)	51 (1.40)	0.030
Sulfonylurea	437 (11.97)	376 (10.30)	0.053	1004 (12.30)	420 (11.49)	0.025
α-glucosidase inhibitors	77 (2.11)	101 (2.77)	0.043	236 (2.89)	115 (3.14)	0.014
Thiazolidinediones	84 (2.30)	75 (2.05)	0.017	219 (2.68)	86 (2.36)	0.020
DPP-4 inhibitors	241 (6.6)	237 (6.49)	0.004	628 (7.69)	266 (7.28)	0.016
SGLT2 inhibitors	17 (0.47)	34 (0.93)	0.056	42 (0.52)	35 (0.96)	0.051
GLP1 agonists	5 (0.14)	5 (0.14)	0.000	10 (0.12)	4.81 (0.13)	0.004
Statin	358 (9.81)	358 (9.81)	0.000	862 (10.57)	398 (10.89)	0.010
Fibrates	83 (2.27)	79 (2.16)	0.007	179 (2.20)	79 (2.17)	0.002
Silymarin	851 (23.31)	883 (24.19)	0.021	1924 (23.57)	910 (24.88)	0.031

Abbreviations: IPTW, inverse probability of treatment weighting; ASMD, absolute standardized mean difference; ETV, Entecavir; TDF/TAF, Tenofovir Disoproxil Fumarate/ Tenofovir Alafenamide Fumarate; HCV, Hepatitis C virus; HDV, Hepatitis D virus; HIV, Hepatitis I virus; EVB, Esophageal varices with bleeding; ACEIs, Angiotensin-converting enzyme inhibitors; ARBs, Angiotensin II receptor blockers; CCBs, Calcium-channel blockers; SGLT2, Sodium-glucose cotransporter-2; GLP1, Glucagon-like peptide-1; y, year.

[a] The absolute standardized mean difference less than 0.1 indicates well-balanced between groups.

eTable 9. Patients' characteristics within PT cohort after propensity score methods for liver transplantation analysis.

Characteristics	Population after PSM			Population after IPTW		
	ETV (n = 1747)	TDF/TAF (n = 1747)	ASMD[a]	ETV (n = 4696)	TDF/TAF (n = 1752)	ASMD[a]
**Mean age (SD), y**	55.14 (11.51)	54.93 (12.40)	0.017	57.09 (11.88)	57.35 (12.57)	0.022
**Gender, n (%)**						
Male	1333 (76.30)	1336 (76.47)	0.004	3558 (75.78)	1331 (75.95)	0.004
Female	414 (23.70)	411 (23.53)	0.004	1137 (24.22)	421 (24.05)	0.004
**Comorbidities, n (%)**						
HCV co-infection	73 (4.18)	72 (4.12)	0.003	219 (4.66)	86 (4.92)	0.012
HDV co-infection	<3	<3	0.000	3 (0.07)	<3	0.002
HEV co-infection	<3	<3	0.000	<3	<3	0.006
HIV co-infection	3 (0.17)	20 (1.14)	0.121	5 (0.11)	21 (1.21)	0.137
Alcoholic cirrhosis	41 (2.35)	37 (2.12)	0.016	91 (1.94)	34 (1.95)	0.001
Biliary cirrhosis	<3	<3	0.211	<3	<3	0.005
Hypertension	512 (29.31)	522 (29.88)	0.013	1627 (34.64)	615 (35.09)	0.009
Hyperlipidemia	278 (15.91)	309 (17.69)	0.047	784 (16.70)	318 (18.17)	0.039
Diabetes	386 (22.10)	380 (21.75)	0.008	1193 (25.41)	449 (25.61)	0.005
Chronic kidney disease	72 (4.12)	63 (3.61)	0.027	273 (5.82)	101 (5.76)	0.003
**History of complications, n (%)**						
Ascites	68 (3.89)	77 (4.41)	0.026	195 (4.15)	70 (4.02)	0.007
Hepatic encephalophy	325 (18.60)	338 (19.35)	0.019	819 (17.43)	318 (18.16)	0.019
EVB	10 (0.57)	17 (0.97)	0.046	72 (1.54)	27 (1.56)	0.001
Hepatorenal syndrome	3 (0.17)	4 (0.23)	0.013	7 (0.14)	<3	0.003
**Charlson Comorbidity Index**						
Mean (SD)	2.21 (2.12)	2.15 (2.06)	0.087	2.31 (2.14)	2.34 (2.17)	0.042
**Disease progression period (y)**	3.69 (2.48)	3.64 (2.37)	0.012	3.11 (2.40)	3.14 (2.29)	0.012
**Treatment gap period (y)**	0.21 (0.71)	0.21 (0.76)	0.001	0.18 (0.65)	0.18 (0.66)	0.001

eTable 9. Patients' characteristics within PT cohort after propensity score methods for liver transplantation analysis. (continued.)

Characteristics	Population after PSM			Population after IPTW		
	ETV (n = 1747)	TDF/TAF (n = 1747)	ASMD[a]	ETV (n = 4696)	TDF/TAF (n = 1752)	ASMD[a]
**Co-medications, n (%)**						
ACEIs/ARBs	346 (19.81)	310 (17.74)	0.053	1091 (23.23)	356 (20.34)	0.070
β -blockers	271 (15.51)	231 (13.22)	0.065	800 (17.04)	267 (15.26)	0.048
Non selective	136 (7.78)	116 (6.64)	0.044	400 (8.51)	136 (7.74)	0.028
Selective	149 (8.53)	126 (7.21)	0.049	452 (9.62)	145 (8.26)	0.048
CCBs	304 (17.40)	259 (14.83)	0.070	942 (20.06)	302 (17.26)	0.072
Diuretics	309 (17.69)	273 (15.63)	0.055	978 (20.82)	326 (18.62)	0.055
Furosemide	126 (7.21)	93 (5.32)	0.078	388 (8.26)	120 (6.87)	0.053
Spironolactone	100 (5.72)	98 (5.61)	0.005	338 (7.19)	124 (7.09)	0.004
Insulin	77 (4.41)	71 (4.06)	0.017	233 (4.96)	80 (4.59)	0.017
Biguanide	259 (14.83)	267 (15.28)	0.013	771 (16.41)	302 (17.23)	0.022
Meglitinide	38 (2.18)	26 (1.49)	0.051	106 (2.25)	36 (2.04)	0.014
Sulfonylurea	182 (10.42)	191 (10.93)	0.017	553 (11.78)	230 (13.10)	0.040
α-glucosidase inhibitors	45 (2.58)	43 (2.46)	0.007	137 (2.91)	52 (2.97)	0.004
Thiazolidinediones	33 (1.89)	39 (2.23)	0.024	97 (2.08)	43 (2.44)	0.024
DPP-4 inhibitors	146 (8.36)	149 (8.53)	0.006	402 (8.55)	176 (10.05)	0.052
SGLT2 inhibitors	21 (1.20)	14 (0.80)	0.040	40 (0.85)	15 (0.83)	0.002
GLP1 agonists	3 (0.17)	3 (0.17)	0.000	9 (0.20)	<3	0.010
Statin	211 (12.08)	189 (10.82)	0.040	581 (12.38)	194 (11.09)	0.040
Fibrates	31 (1.77)	32 (1.83)	0.004	102 (2.16)	30 (1.73)	0.031
Silymarin	323 (18.49)	382 (21.87)	0.084	892 (19.00)	389 (22.21)	0.080

Abbreviations: IPTW, inverse probability of treatment weighting; ASMD, absolute standardized mean difference; ETV, Entecavir; TDF/TAF, Tenofovir Disoproxil Fumarate/ Tenofovir Alafenamide Fumarate; HCV, Hepatitis C virus; HDV, Hepatitis D virus; HIV, Hepatitis I virus; EVB, Esophageal varices with bleeding; ACEIs, Angiotensin-converting enzyme inhibitors; ARBs, Angiotensin II receptor blockers; CCBs, Calcium-channel blockers; SGLT2, Sodium-glucose cotransporter-2; GLP1, Glucagon-like peptide-1; y, year.

[a] The absolute standardized mean difference less than 0.1 indicates well-balanced between groups.

eTable 10. Clinical outcomes within PT patients after propensity score methods.

Panel A. Population After PSM								
**Outcome**[a]	Patients, n	Events, n	PY	Rate[b] (95% CI)	cHR[c] (95% CI)	p value	aHR[d] (95% CI)	p value
**Composite outcome**								
Tenofovir	1724	401	5648	7.10 (6.42-	0.81 (0.71-0.93)	0.0021	0.82 (0.71-0.94)	0.0033
Entecavir	1724	509	6092	8.36 (7.65-	1.00		1.00	
**Hepatocellular carcinoma**								
Tenofovir	1737	222	6952	3.19 (2.79-3.64)	0.61 (0.49-0.76)	<0.0001	0.60 (0.48-0.75)	<0.0001
Entecavir	1737	354	7451	4.75 (4.27-5.27)	1.00		1.00	
**Death**								
Tenofovir	1762	306	6201	4.93 (4.40-5.52)	0.88 (0.76-1.03)	0.1115	0.93 (0.79-1.08)	0.3374
Entecavir	1762	370	6825	5.42 (4.88-6.00)	1.00		1.00	
**Liver transplantation**								
Tenofovir	1747	48	6969	0.69 (0.51-0.91)	1.27 (0.81-2.00)	0.2948	1.17 (0.73-1.89)	0.5112
Entecavir	1747	37	7773	0.48 (0.34-0.66)	1.00		1.00	

PSM: Propensity score matching; PY: Person-year; cHR: Crude Hazard Ratio; aHR: Adjusted Hazard Ratio.

[a] Patients who had already encountered the relevant outcome before the index date were excluded in every outcome analysis.

[b] Rate was determined by dividing the number of events by the total person-years and presented as per 100 person-years.

[c] Crude HR was calculated by subdistribution COX proportional hazards model.

[d] Adjusted HR was calculated by subdistribution COX proportional hazards model adjusted for all variables.

eTable10. Clinical outcomes within PT patients after propensity score methods. (continued.)

Panel B. Population After IPTW								
**Outcome**[a]	Patients, n	Events, n	PY	Rate[b] (95% CI)	cHR[c] (95% CI)	p value	aHR[d] (95% CI)	p value
**Composite outcome**								
Tenofovir	1727	459	5715	8.03 (7.31-	0.81 (0.73-0.90)	0.0001	0.81 (0.73-0.90)	0.0001
Entecavir	4611	1588	16765	9.47 (9.01-	1.00		1.00	
**Hepatocellular carcinoma**								
Tenofovir	1738	246	7240	3.40 (2.99-3.85)	0.62 (0.51-0.74)	<.0001	0.60 (0.50-0.72)	<.0001
Entecavir	4633	1064	21529	4.94 (4.65-5.25)	1.00		1.00	
**Death**								
Tenofovir	1764	369	6319	5.84 (5.26-6.47)	0.91 (0.81-1.02)	0.1001	0.91 (0.81-1.03)	0.1326
Entecavir	4717	1190	19399	6.13 (5.79-6.49)	1.00		1.00	
**Liver transplantation**								
Tenofovir	1752	45	7324	0.61 (0.45-	1.33 (0.92-	0.126	1.23 (0.85-	0.2731
Entecavir	4696	99	22989	0.43 (0.35-	1.00		1.00	

IPTW: Inverse probability of treatment weighting; PY: Person-year; cHR: Crude Hazard Ratio; aHR: Adjusted Hazard Ratio.

[a] Patients who had already encountered the relevant outcome before the index date were excluded in every outcome analysis.

[b] Rate was determined by dividing the number of events by the total person-years and presented as per 100 person-years.

[c] Crude HR was calculated by subdistribution COX proportional hazards model.

[d] Adjusted HR was calculated by subdistribution COX proportional hazards model adjusted for all variables.

eTable 11. Analyses for negative control outcome.

Negative control outcome	Population after PSM		Population after IPTW	
	asHR (95% CI)	p value	asHR (95% CI)	p value
**Myocardial infarction**				
**PUT subcohort**				
Tenofovir	1.01 (0.63-1.62)	0.9753	1.01 (0.69-1.46)	0.969
Entecavir	1.00 (Reference)		1.00 (Reference)	
**PT subcohort**				
Tenofovir	1.08 (0.52-2.26)	0.84	1.15 (0.69-1.94)	0.589
Entecavir	1.00 (Reference)		1.00 (Reference)	

Abbreviations: asHR, adjusted sub-distribution hazard ratio

Figures

eFigure 1. Cumulative incidence curves for TDF/TAF users versus ETV users within PUT cohorts after IPTW. (A) Composite outcome (B) Hepatocellular carcinoma (C) Death (D) Liver transplantation.

(A)

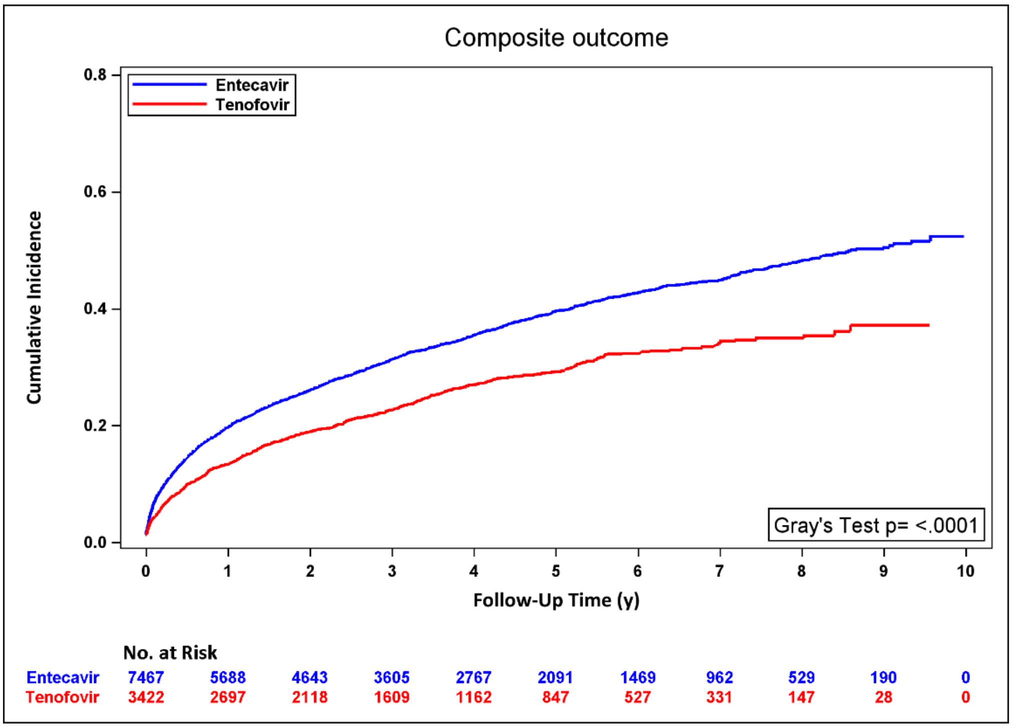

(B)

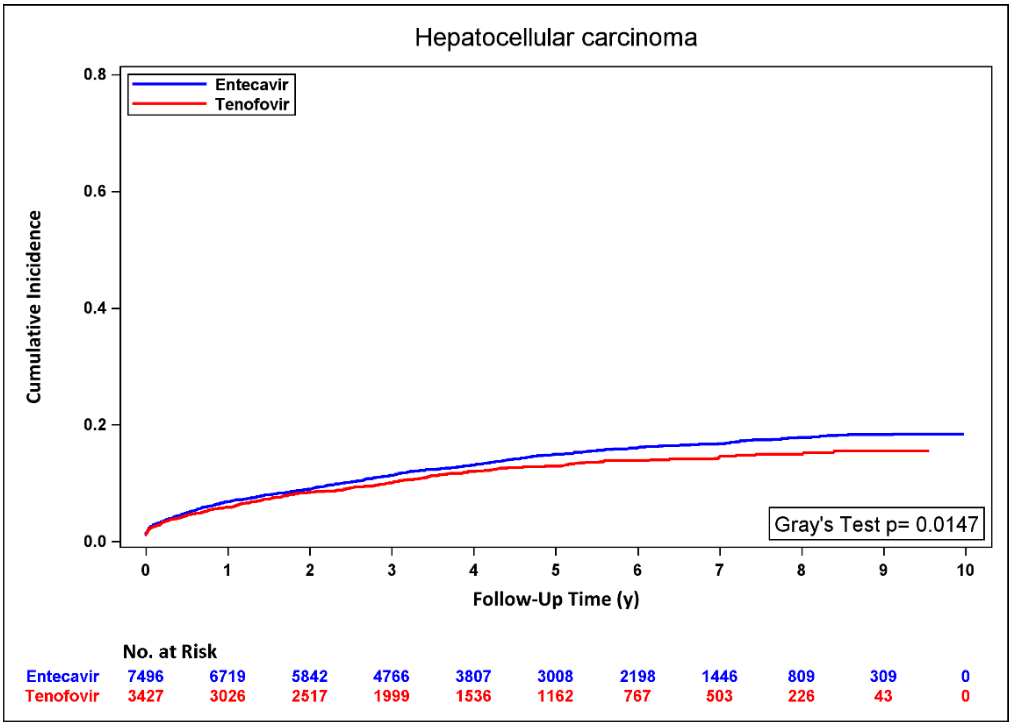

(C)

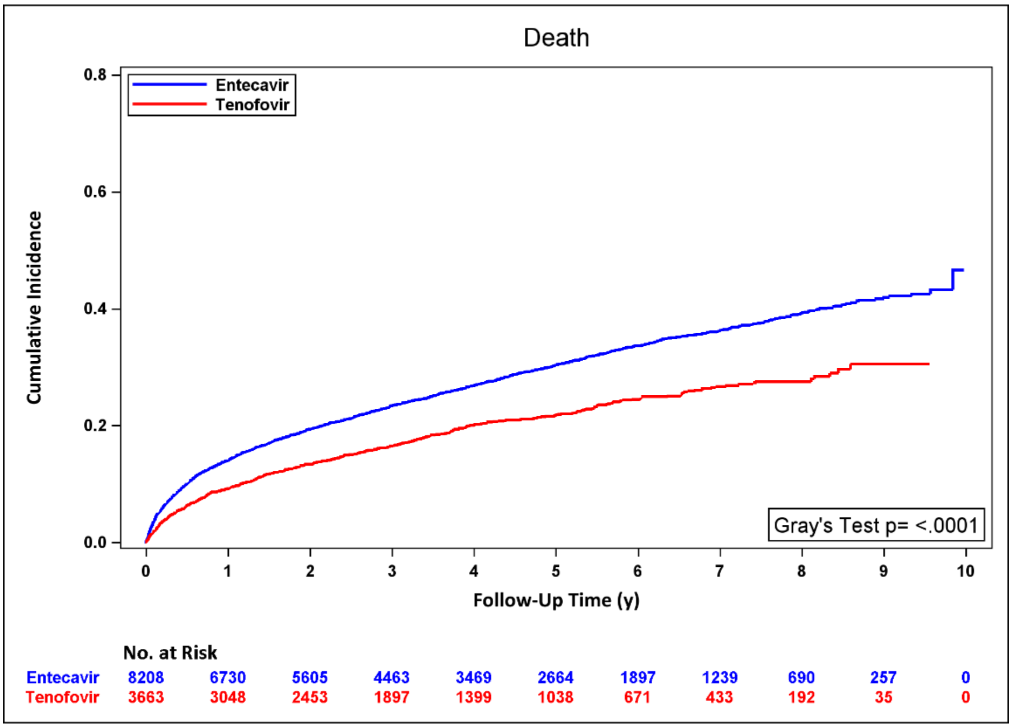
Death
Entecavir
Tenofovir
Gray's Test p= <.0001
Cumulative Inicidence
0.0
0.2
0.4
0.6
0.8
0 1 2 3 4 5 6 7 8 9 10
Follow-Up Time (y)
No. at Risk
Entecavir 8208 6730 5605 4463 3469 2664 1897 1239 690 257 0
Tenofovir 3663 3048 2453 1897 1399 1038 671 433 192 35 0

(D)

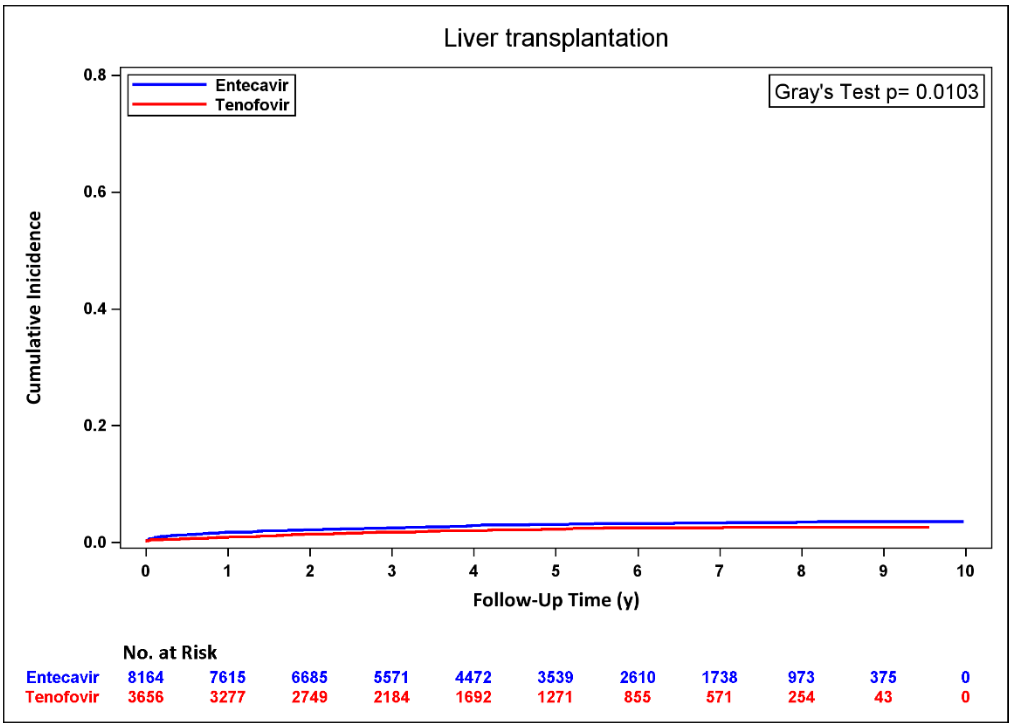
Liver transplantation
Entecavir
Tenofovir
Gray's Test p= 0.0103
Cumulative Inicidence
0.0
0.2
0.4
0.6
0.8
0 1 2 3 4 5 6 7 8 9 10
Follow-Up Time (y)
No. at Risk
Entecavir 8164 7615 6685 5571 4472 3539 2610 1738 973 375 0
Tenofovir 3656 3277 2749 2184 1692 1271 855 571 254 43 0

eFigure 2. Cumulative incidence curves for TDF/TAF users versus ETV users within PT cohorts after IPTW. (A) Composite outcome (B) Hepatocellular carcinoma (C) Death (D) Liver transplantation.

(A)

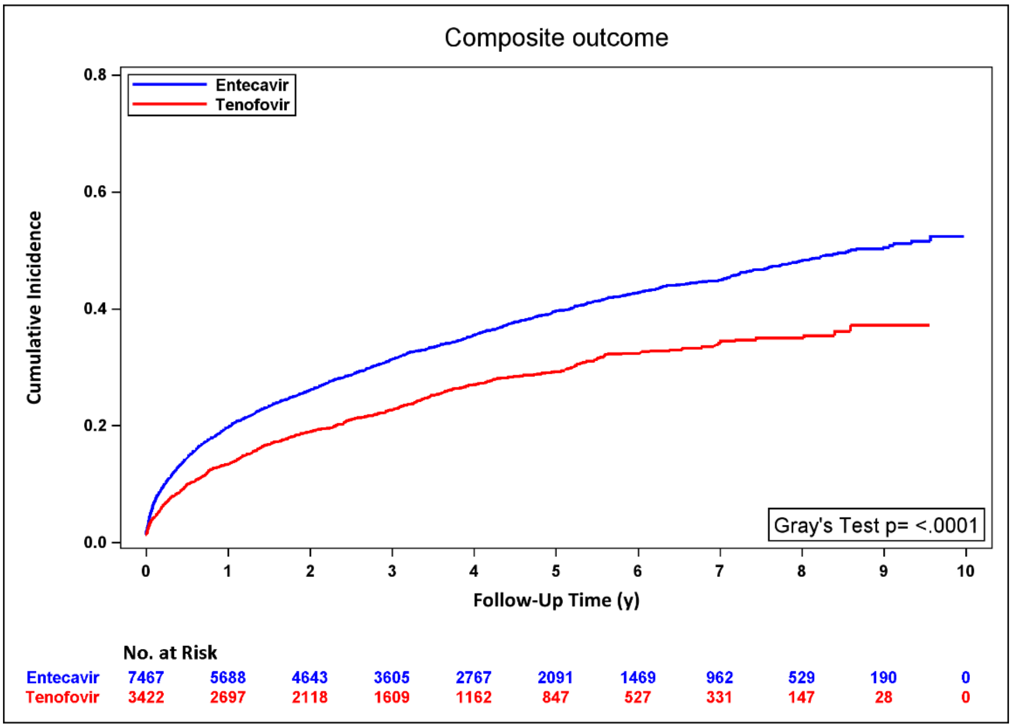

(B)

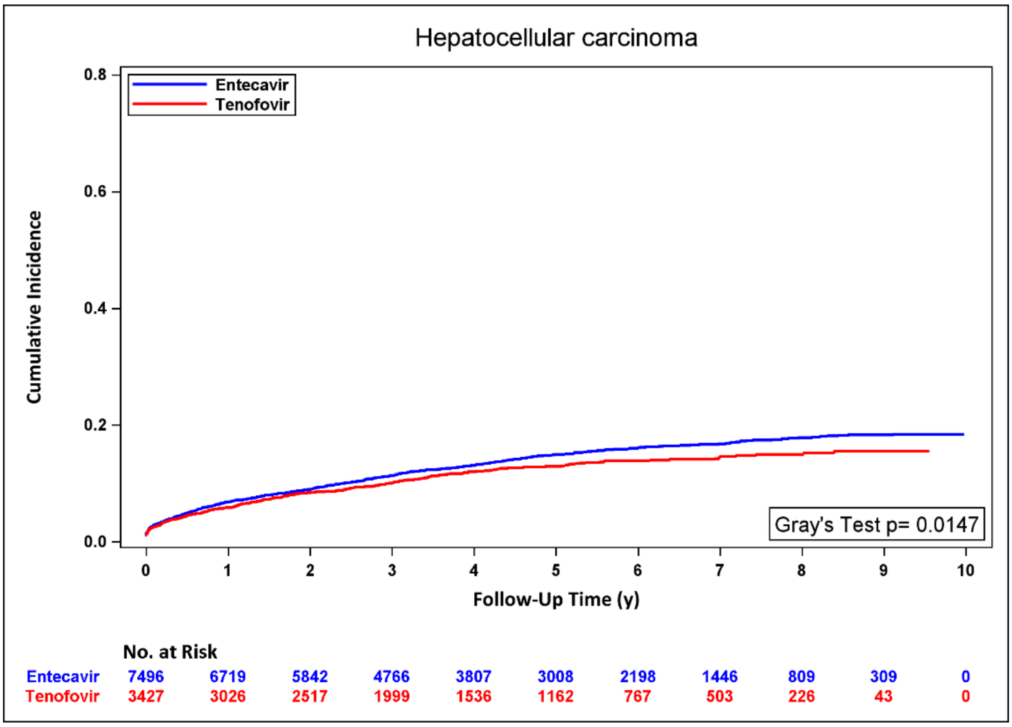

(C)

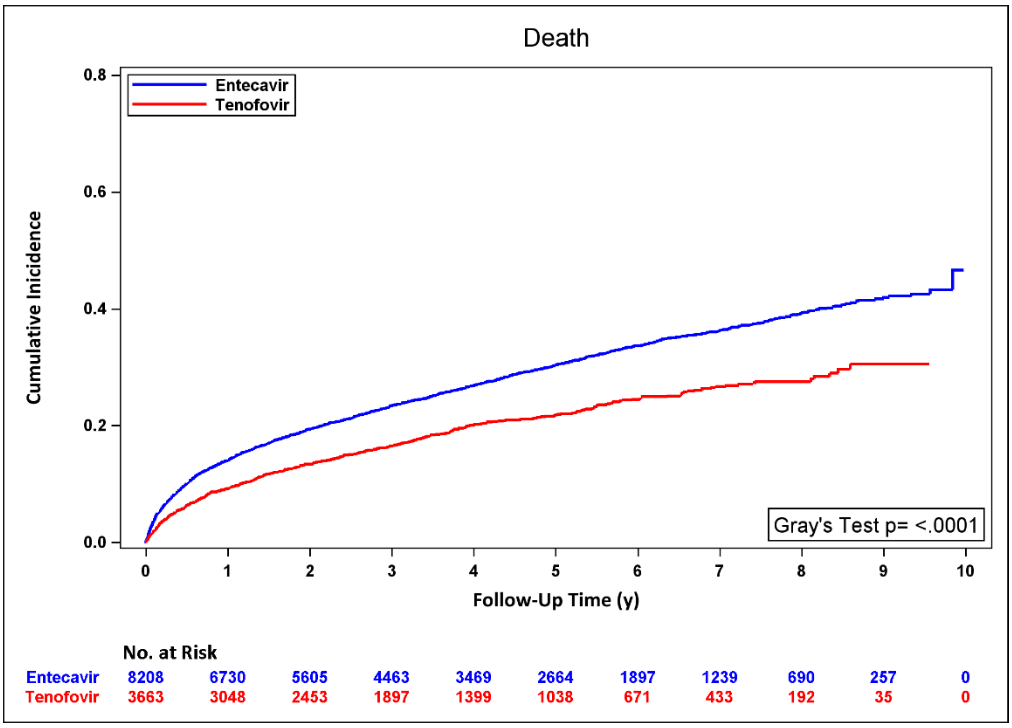
Death
Entecavir
Tenofovir
Gray's Test p= <.0001
Cumulative Incidence
0.0
0.2
0.4
0.6
0.8
0 1 2 3 4 5 6 7 8 9 10
Follow-Up Time (y)
No. at Risk
Entecavir 8208 6730 5605 4463 3469 2664 1897 1239 690 257 0
Tenofovir 3663 3048 2453 1897 1399 1038 671 433 192 35 0

(D)

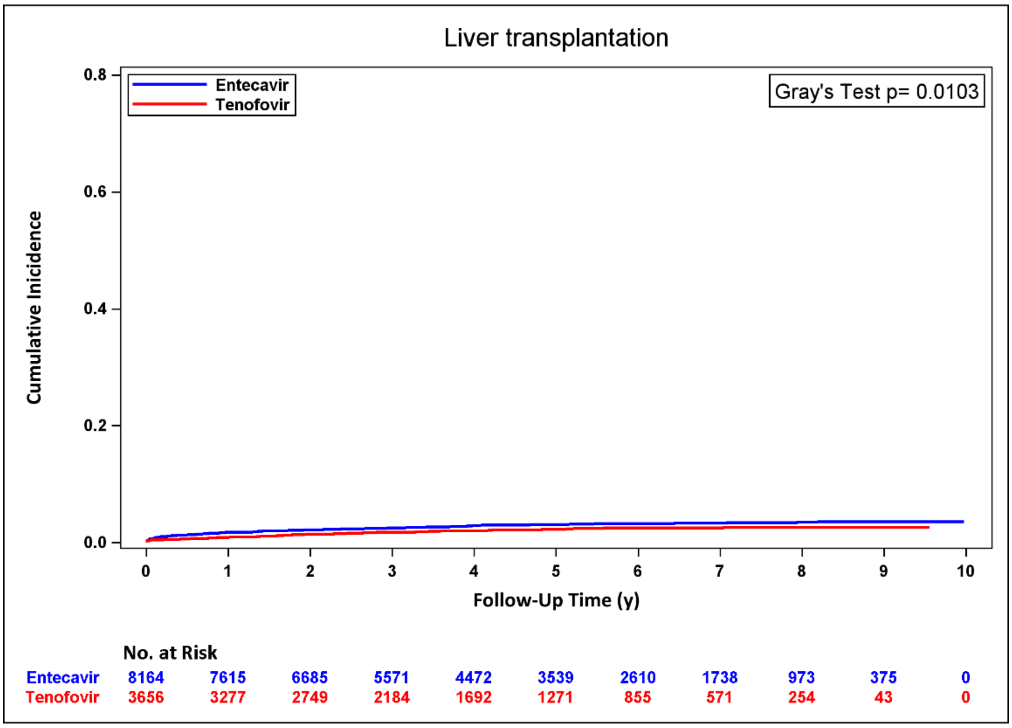
Liver transplantation
Entecavir
Tenofovir
Gray's Test p= 0.0103
Cumulative Incidence
0.0
0.2
0.4
0.6
0.8
0 1 2 3 4 5 6 7 8 9 10
Follow-Up Time (y)
No. at Risk
Entecavir 8164 7615 6685 5571 4472 3539 2610 1738 973 375 0
Tenofovir 3656 3277 2749 2184 1692 1271 855 571 254 43 0
